# Flexible Strain Sensors with Ultra-High Sensitivity and Wide Range Enabled by Crack-Modulated Electrical Pathways

**DOI:** 10.1007/s40820-024-01571-6

**Published:** 2024-11-18

**Authors:** Yunzhao Bai, Yunlei Zhou, Xuanyu Wu, Mengfei Yin, Liting Yin, Shiyuan Qu, Fan Zhang, Kan Li, YongAn Huang

**Affiliations:** 1 State Key Laboratory of Intelligent Manufacturing Equipment and Technology, Huazhong University of Science and Technology, Wuhan, 430074 People’s Republic of China; 2 Flexible Electronics Research Center, Huazhong University of Science and Technology, Wuhan, 430074 People’s Republic of China; 3 Hangzhou Institute of Technology, Xidian University, Hangzhou, 311200 People’s Republic of China; 4 School of Mechano-Electronic Engineering, Xidian University, Xi’an, 710071 People’s Republic of China; 5 Department of Aerospace and Mechanical Engineering, University of Southern California, Los Angeles, CA 90089 USA

**Keywords:** Flexible strain sensor, Fabric, Crack, Response regulation, Epidermal device

## Abstract

This study presents a breakthrough in flexible strain sensor technology with the development of an ultra-high sensitivity and wide-range sensor, addressing the critical challenge of reconciling sensitivity with measurement range. Inspired by the structure of bamboo slips, we introduce a novel approach that utilises liquid metal to modulate the electrical pathways within a cracked platinum fabric electrode. The resulting sensor demonstrates a gauge factor greater than 10^8^ and a strain measurement capability exceeding 100%. The integration of patterned liquid metal enables customisable tuning of the sensor’s response, while the porous fabric structure ensures superior comfort and air permeability for the wearer. Our design not only optimises the sensor’s performance but also enhances the electrical stability that is essential for practical applications. Through systematic investigation, we reveal the intrinsic mechanisms governing the sensor’s response, offering valuable insights for the design of wearable strain sensors. The sensor’s exceptional performance across a spectrum of applications, from micro-strain to large-strain detection, highlights its potential for a wide range of real-world uses, demonstrating a significant advancement in the field of flexible electronics.

**Supplementary Information:**

The online version contains supplementary material available at 10.1007/s40820-024-01571-6.

## Highlights

- A method is proposed for the modulation of electrical pathways in the cracks of stretchable electrodes using liquid metals, based on which ultra-high sensitivity (> 10^8^) and large-range (> 100%) strain sensors are realised.
- A secondary modulation of the response (or performance) of the sensor is proposed, allowing not only electrical modulation by liquid metal patterning during fabrication, but also mechanical modulation by pre-stretching at the time of use.
- The air permeability and stability of the patterned liquid metal electrode region is optimised to enable air permeability similar to that of conventional fabrics and cycle durability in excess of 2000 cycles.

**Supplementary Information:**

The online version contains supplementary material available at 10.1007/s40820-024-01571-6.

## Graphical abstract

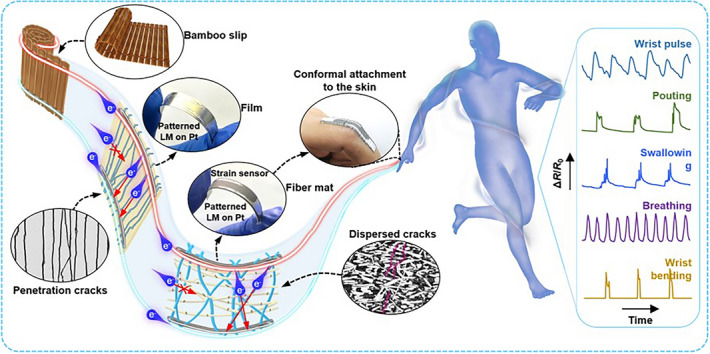

## Introduction

The explosion of flexible electronics has brought about major changes in many areas of technology and life [1–4], such as wearable electronics [5–11], smart electronic skins for aircrafts and robots [12, 13], microair vehicles [14], efficient energy harvesting and storage devices [15, 16], human–computer interaction technologies [6–8, 17–19] and micro-optoelectronic devices [20–22]. Resistive strain sensors, characterised by their notable sensitivity and simplistic structure and readout circuitry, are widely employed in wearable strain sensing devices for human motion signal monitoring facilitates precise detection of various signals, encompassing joint motion [23, 24], voice [6, 23], expression [7], respiration [25–27] and pulse [28–30]. To acquire signals ranging from weak pulse signals to large-strain joint motion signals without affecting normal human activities, flexible strain sensors with high sensitivity and wide range are required. However, increasing the sensitivity of a device may come at the cost of reducing its range, and vice versa. This is because increasing sensitivity accelerates the depletion of the electroactive material, whereas increasing the range decelerates it [31].

In order to enhance the sensitivity of strain sensors, a large number of previous studies have reported crack-based resistive strain sensors to obtain high sensitivity. Changing the rate of electrode cracking and the morphology of the crack during stretching is an effective means of modulating the response of resistive strain sensors, which can be carried out through the structural design of the sensors and the updating of the material system [32]. In an effort to further boost sensitivity, a variety of structural designs have been investigated [33], including slit structures that mimic the sensory functions of scorpions and spiders [34, 35], and metamaterials featuring a negative Poisson’s ratio [31]. Despite these advancements, the measurement range of these high-sensitivity sensors remains constrained. To accommodate the need for a more extensive range, researchers have examined the possibility of altering the behaviour of electroactive materials undergoing penetrating cracks by layering two-dimensional materials beneath the cracked material [36]. Additionally, the incorporation of pleated or meandering structures has been proposed to mitigate the direct stress response of the electroactive material, thus slowing down its rate of dissipation [37, 38]. However, introducing a variety of materials can elevate the risk of sensor failure due to discrepancies in mechanical properties, and the complexity of pleated and meandering designs may complicate the manufacturing process, potentially impeding commercial viability [31, 39, 40].

Liquid metal has excellent tensile and conductive capabilities with a wide range of applications in the field of stretchable electrodes, including interconnecting leads, self-healing conductors [41–43] and strain sensors. In pursuit of a broader measurement range, some research has shifted its focus from crack-based strain sensors to liquid metals [44–46]. Characterised by superior tensile properties that avert stress mismatches within the material system, liquid metal-based strain sensors are capable of reaching ranges in excess of 500% [46, 47]. Nonetheless, such a broad range is overkill for the detection of human strain signals, which are generally less than 100% [5], and is accompanied by a diminished sensitivity that struggles to capture faint signals, such as pulses. Moreover, the superior conductivity of liquid metals leads to a reduced base resistance in the sensitive unit, amplifying the strain response of the leads and potentially introducing a multitude of invalid signals into the strain measurement process. Given that high sensitivity often comes with a limited range and that a broader range is often paired with lower sensitivity, the quest for developing strain sensors that are both highly sensitive and have a wide measurement range for human applications continues to be a significant area of research.

The present study draws inspiration from the historical artefact of bamboo slips, which are composed of rigid strips bound together by the interlacing of flexible strands, creating a cohesive and scrollable entity. This transformation adapts the naturally rigid bamboo into a more pliable form, endowing it with the capability for facile bending and curling. The enhanced adaptability of the bamboo slips augments their storability, making them amenable to straightforward archiving and transportation processes. Building upon the ancient technique of bamboo slip construction, we propose a method where the strategic placement of flexible cords along the opposing edges of individual bamboo slats facilitates the assembly into a unified structure. In a metaphorical application of this concept, liquid metal is utilised as a “rope” within our research to effectively “lock” the edges of fissures within the metallic layer of stretchable electrodes. Edge-locking liquid metal connects the dispersed electrical fragments together to regain a smooth conductive path for the whole. This innovative strategy enables the reconfiguration of electrical pathways from a direction parallel to the axis of elongation to one that is perpendicular to it, thereby dynamically modifying the conductive network in response to mechanical deformation. Distinguishing from the traditional strategy of changing the dispersion rate of electroactive materials by altering the material [48–51] or structure [52] of the sensor, this work proposes a novel strategy of altering the cracking electrical pathway of established electroactive materials, which is able to effectively modulate the response of flexible resistive strain sensors. This strategy is applicable to a wide range of elastic materials, and a thermoplastic polyurethanes (TPU, Tecoflex™ SG-80A by Lubrizol, France) is used in this work due to its remarkable ductility (i.e. failure strain > 0.5) [53–55].

This study investigates the mechanical response of stretchable electrodes under strain, with a focus on liquid metal edge-locking effects on platinum fissures within thermoplastic polyurethane (TPU) substrates. Utilising the high sensitivity of TPU fibre mats, we developed stretchable strain sensors and explored the impact of geometrical configurations of liquid metal patterning on their performance. Our findings reveal that through careful optimisation, the operational range of these sensors can exceed 110% without sacrificing sensitivity, as demonstrated by a Gauge Factor over 10^8^. These sensors are capable of detecting a comprehensive range of strain measurements, from microstrain to large strain, suitable for various epidermal strain measurement scenarios. The straightforward and effective nature of our electrical modulation technique not only simplifies the development process but also bolsters the prospects of commercialising this sophisticated strain-sensing technology.

## Experimental Section

### Materials

Thermoplastic polyurethane (TPU, Tecoflex™ SG-80A) was purchased from Lubrizol. Tetrahydrofuran (THF) and N, N-dimethylformamide (DMF) were purchased from Aladdin. Platinum (Pt) magnetron sputtering targets were purchased from Suzhou Research Materials Microtechnology. The liquid metal (LM) was purchased from Shenyang Jiabei Commerce Ltd with a mass fraction ratio of 75% gallium and 25% indium.

### Preparation of TPU Fibre Mat

TPU fibre mats were produced under certain spinning condition (25 ± 3 °C and 45 ± 5%) by using an electrospinning machine (lab-built). The substrate movement path and spinning time can be adjusted according to the required fibre mat area and thickness. The solution feed rate and applied voltage were set at 10 μL h^−1^ and 7.5 kV. The nanofibres were continuously collected on a paper-covered flat aluminium plate at a distance of 15 cm. The resultant samples were finally placed in a vacuum oven under 70 °C for 2 h to remove the residual solvent.

### Preparation of Metal Electrodes

Platinum electrodes were prepared by magnetron sputtering process deposition with a thickness of 50 nm. Liquid metal electrodes were prepared by using laser-cut stainless steel (50 μm) as a mask, and liquid metal was applied to the target surface using a small brush.

### Characterisation

Electron microscope (SU3900, HITACHI) was used to observe the morphology of platinum-plated TPU films and platinum-plated TPU fibre mats. Mechanical tests and electrical properties of sensors were performed using a universal testing machine (5944, Instron) and a multimeter (34465A, Keysight), respectively. There were two conductive tapes as electrodes at both ends of the sensor to connect with the multimeter for signal collection. The humidity stability of the sensor was carried out in a large clear plastic bag, and increasing humidity was acquired by injecting humidity into the bag using a humidifier. A programmable constant temperature and humidity test chamber (Bohangda Instruments, China) were used to detect the water vapour permeability of the sensor.

## Results and Discussion

### Ultra-High Sensitivity, Large-Range Fabric Strain Sensors

**Fig. 1:**
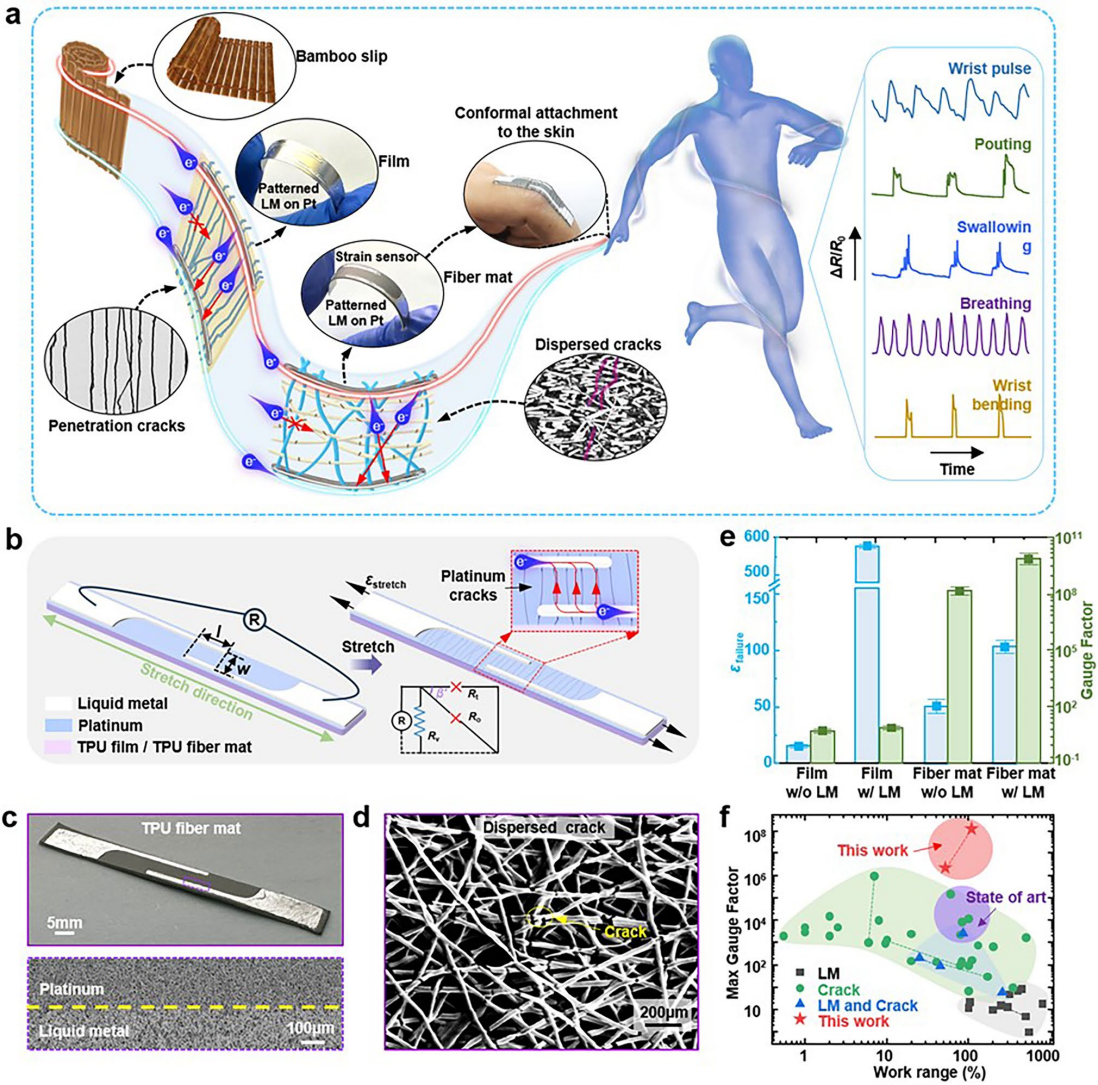
Ultra-high sensitivity, large-range fabric strain sensors inspired by ancient Chinese bamboo slips. **a** Schematic illustration of the sensor and its potential applications. **b** Three-dimensional structure of the sensor and schematic diagram of platinum-plated TPU film or TPU fibre mat after stretching. **c** Optical image of a platinum-plated TPU fibre mat coated with edge-locking liquid metal on a release paper (top). SEM image of the edge of the patterned liquid metal locally enlarged (bottom). **d** SEM image of the platinum-plated TPU fibre mat at transverse strain *ε*_stretch_ = 0.3. **e** Electrical failure strain and maximum Gauge Factor of four different electrodes: platinum-plated TPU film, platinum-plated TPU fibre mat, platinum-plated TPU film coated with edge-locking liquid metal and platinum-plated TPU fibre mat coated with edge-locking liquid metal. The spacing of the liquid metal was: *w* = 3 mm, and the overlap length: *l* = 10 mm. **f** Comparison of the performance of the fabric strain sensor proposed in this work with other crack or liquid metal-based sensors in literature

Inspired by the structure of ancient bamboo slips, we designed a crack-based flexible strain sensor as presented in Fig. 1a. During the tensile deformation process, the metal layer develops perforations, termed dispersed bamboo strips. Liquid metals emerge as a superior choice for stretchable electrodes due to their exceptional ductility, consistent electrical conductivity across a range of strain conditions, and a null Young’s modulus that minimally perturbs the system’s inherent mechanical properties. In this context, liquid metal acts as a metaphorical soft cord, bridging the gaps within the fractured metal layer. The targeted application of liquid metal to reestablish electrical connectivity across the fissured metal on the substrate—referred to as the “liquid metal locking edge”—enables charge transport along the perforated metal layer, effectively restoring conductivity. Building on this concept, we have refined the design by substituting the elastic film with an elastic fibre mat, thereby altering the trajectory of the electrical conduction of the cracked electrode. This innovation culminates in the development of a large-range fabric strain sensor with ultra-high sensitivity, capable of adhering seamlessly to the skin surface for comprehensive detection of strain signals across the human body.

Figure 1b shows a schematic of the 3D structure of a flexible electrode reinforced with edge-locked liquid metal and a schematic of its cracking in tension. This structure comprises a lower layer of an elastic thermoplastic polyurethane (TPU) film or TPU fibre mat, a middle layer of a metallic electrode made of platinum and an upper layer of patterned liquid metal. The inset shows the equivalent circuit diagram of the flexible electrode after cracking. Figure S1a, c presents optical patterns (top) and scanning electron microscopy (SEM) images (bottom) of the flexible electrodes, which are formed by integrating edge-locking liquid metal with platinum-plated TPU films and TPU fibre mats placed on release paper, respectively. Figure S1b, d displays SEM images of surface platinum cracks on platinum-plated TPU films and TPU fibre mats, respectively, under strain of 0.3. After an initial phase of elastic–plastic deformation during stretching, parallel penetrating cracks rapidly emerge, leading to the swift failure of electrodes composed solely of platinum-plated TPU films. Conversely, the platinum electrode layer on TPU fibre mats progressively develops cracks throughout the stretching process. These cracks effectively enhance the failure strain compared to the penetrating straight cracks.

The electrical failure strains will be regulated with the addition of edge-locking liquid metal. Figure 1e presents a comprehensive comparison of the flexible electrodes’ responses to strain, with platinum-plated TPU films and TPU fibre mats, before and after the integration of edge-locking liquid metal. The pure platinum-plated TPU film electrodes experience complete failure at a tensile strain of approximately 0.15. In contrast, the platinum-plated TPU film electrode with edge-locking liquid metal exhibits a low Gauge Factor at large strains, enduring up to more than 500% during tensile stretching. The electrical failure strains for the platinum-plated TPU fibre mat electrodes before and after the application of edge-locking liquid metal are approximately 0.5 and 0.75, respectively. This indicates that the incorporation of liquid metal can effectively modulate the strain response of the platinum-plated fibre mat-based electrodes.

Both before and after the application of edge-locking liquid metal, the max Gauge Factors of the platinum-plated TPU fibre mat electrodes exceed 10^8^, suggesting their potential as high-sensitivity strain sensors. The liquid metal-covered area on the strain sensor retains a porous structure, as shown in Fig. S2. This feature effectively preserves the breathability of the fibre mat, ensuring comfort during wear, as detailed in Section “Vapour permeability”. A comparison of the range and max Gauge Factor of crack-based and liquid metal-based flexible strain sensors from the literature is presented in Fig. 1f. A systematic study, described in detail in the subsequent sections, demonstrates that these sensors surpass the highest available Gauge Factor and exhibit the highest sensitivity within the wearable range.

### Mechanical Analysis

**Fig. 2:**
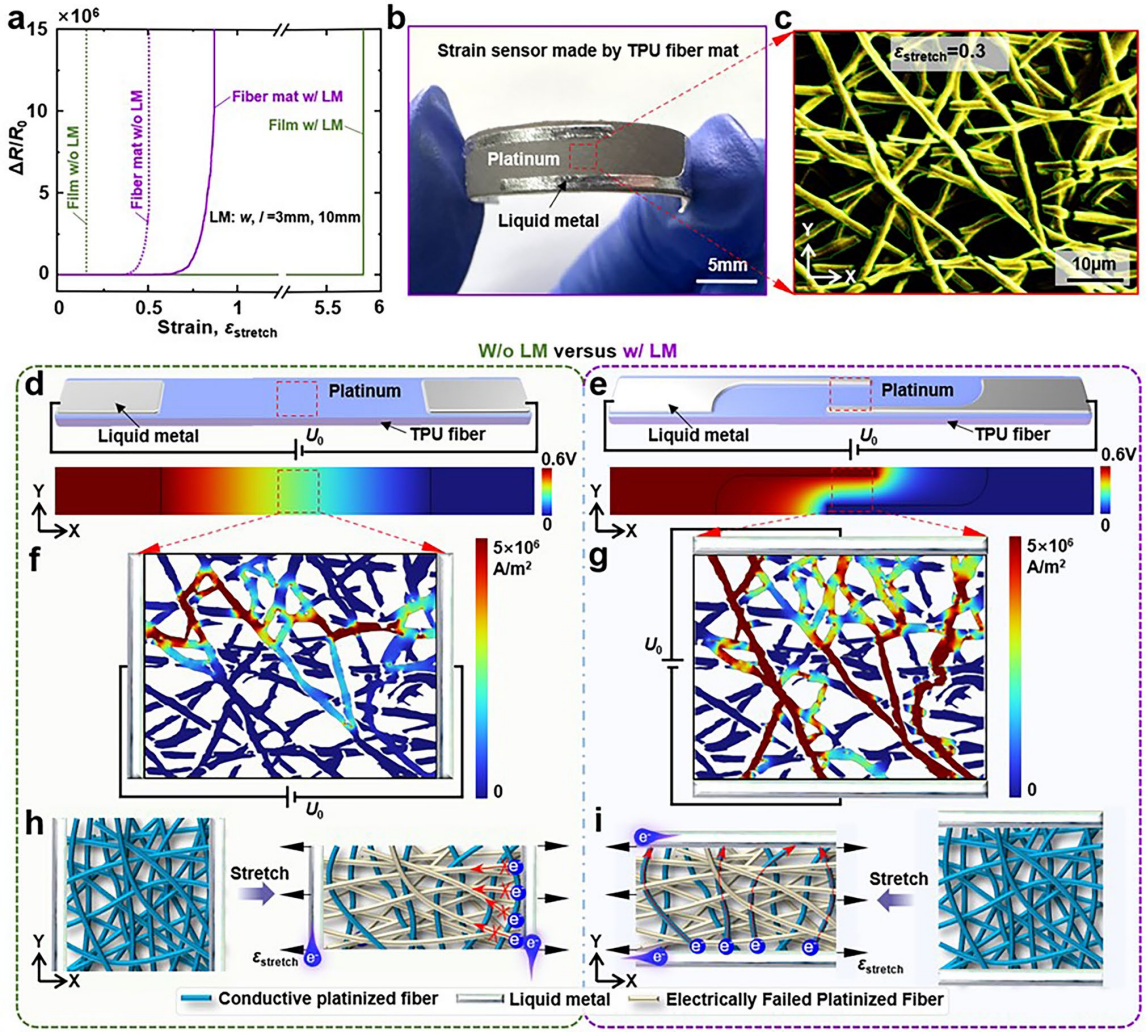
Working mechanism for the strain sensor of platinum-plated TPU fibre mats coated with edge-locking liquid metal. **a** Response of four different electrodes: platinum-plated TPU film, platinum-plated TPU fibre mat, platinum-plated TPU film coated with edge-locking liquid metal and platinum-plated TPU fibre mat coated with edge-locking liquid metal. The spacing of the liquid metal was: *w* = 3 mm, and the overlap length: *l* = 10 mm. **b** Optical photograph of platinum-plated TPU fibre mat coated with edge-locking liquid metal on release paper as strain sensor. **c** Magnification of the red dashed box part of the SEM image in Fig. S4 at the strain of 0.3. The green line in Fig. shows the edge of the electrode. **d** and **e** are cloud diagrams of the electric field distribution of the platinum-plated TPU fibre mat electrodes before (**d**) and after (**e**) coated with edge-locking liquid metal, respectively. **f** and **g** are the current density cloud plots after applying the potential *U*_0_ along the X and Y directions, respectively. **h** and **i** are schematic diagrams of the electrical failure of the fibres and the electrode electrical pathways throughout the platinum-plated TPU fibres before and after the application of the edge-locking liquid metal, respectively, when subjected to tension

Figure 2a presents a comparative analysis of the performance of four distinct types of electrodes: platinum-plated TPU film, platinum-plated TPU fibre mat, platinum-plated TPU film augmented with edge-locking liquid metal and platinum-plated TPU fibre mat coated with edge-locking liquid metal. The parameters for the edge-locking liquid metal were a spacing of w = 3 mm and an overlap length of l = 10 mm. In light of the findings and the requirement for wearable strain sensors to exhibit high sensitivity over a broad range, the platinum-plated TPU fibre mat electrodes integrated with liquid metal featuring an edge-locking structure were selected for use as piezoresistive strain sensors. Figure 2b displays an optical image of the strain sensor when bent on release paper.

For piezoresistive strain sensors, the variation in resistance in response to strain is a direct indicator of their efficacy. This alteration in resistance is influenced by the combined mechanical and electrical attributes of the sensor itself. Mechanically, as the sensor experiences tensile strain, the electroactive layer undergoes deformation until it fractures, leading to a consequent change in the sensor’s resistance. Electrically, beyond the mechanical deformation and crack formation, the selection of the conductive pathway is also a significant factor affecting the resistance response to strain. To thoroughly comprehend the operating principle of the proposed strain sensor and to achieve modulation of the response, a systematic examination of the sensor’s electrical properties is essential.

The strain behaviour of fibres coated with electroactive material in a fibre mat-based piezoresistive strain sensor dictates the alteration of its electrical traits. To this end, the microscopic fibre strain behaviour at various angles under macroscopic tensile strain was scrutinised. A straightforward model was proposed to understand the deformation of a specific fibre on a strain sensor when subjected to tensile strain. Figure S3a, b illustrates the deformation of a straight fibre segment under tensile strain at the substrate. With the fibre segment’s initial length denoted as *L* and the inclination angle as *α*_0_, and assuming linear elasticity, the strain applied to the substrate is *ε*_*x*_. Given that the fibres’ characteristic dimensions are significantly smaller than those of the fibre mat substrate, the impact of fibre stiffness on the substrate’s deformation is disregarded. Accounting for the Poisson’s ratio effect in the substrate’s transverse direction, the strain within the straight fibre section can be articulated as: $\varepsilon_{{{\text{fiber}}}} = \sqrt {\left[ {(1 + \varepsilon_{{\text{x}}} )\cos \alpha_{0} } \right]^{2} + \left[ {(1 - \nu_{{{\text{fibermat}}}} \varepsilon_{{\text{x}}} )\sin \alpha_{0} } \right]^{2} } - 1$, where *ν*_fibremat_ is the Poisson’s ratio of the fibre mat substrate. It is evident from the equation that as the angle *α*_0_ diminishes, the fibres undergo a higher strain during the substrate stretching process. Furthermore, the strain behaviour of the TPU fibre network was simulated using the finite element analysis software ABAQUS (refer to Note S1 and Figs. S4 and S5 for detailed information), with the outcomes aligning with the theoretical analysis. Consequently, as the tensile strain continues to increase, new angles of platinum cracks emerge on the fibres, enabling the electrodes to maintain a response under persistent tensile strain. It is this ongoing generation of Pt cracks within the conductive pathway that leads to a progressive and significant alteration in the sensor’s resistance value, thereby facilitating the achievement of high sensitivity.

Figure S6 presents the SEM images of the platinum-plated TPU fibre mats along the X-axis for various strains *ε*_stretch_ = 0, 0.1, 0.3, 0.5 and 1. The region within the red dashed box of the SEM image showing the platinum electrode crack on the TPU fibre mat with *ε*_stretch_ = 0.3 in Fig. S6 is extracted and displayed in Fig. 2c. It is observed that the platinum layer on the fibres aligned along the X-axis is prone to premature cracking, leading to electrical failure under X-axis tensile loading. In contrast, the platinum layer on the fibres oriented along the Y-axis can withstand larger applied strains, which corroborates the theoretical analysis. Unlike the platinum-plated TPU films, which continuously generate parallel penetration-type cracks in the platinum layer during stretching (as shown in Fig. S7a), the interwoven fibre structure at the microlevel prevents the propagation of cracks to adjacent fibres. This characteristic helps avoid the continuous cracking of the platinum layer and the rapid failure of the platinum-plated TPU fibre mat electrodes during stretching, explaining why the failure strain in platinum-plated TPU fibre mats is significantly higher than in platinum-plated TPU films.

To delve into the impact of the edge-locking liquid metal, the electrical characteristics of the platinum-plated TPU fibre mat electrodes before and after the incorporation of the edge-locking liquid metal were examined through simulation and comparison using the commercial finite element analysis software COMSOL. The calculated voltage distributions after applying a voltage drop across the liquid metal on both sides of the stretchable electrode are depicted in Fig. 2d, e. The direction of the voltage drop with the addition of the liquid metal on the locking edge shifts from primarily along the X-axis to mainly along the Y-axis. Moreover, the effective area subjected to the voltage drop transitions from the entire electrode to the region surrounding the overlapped length of the liquid metal in the X-axis direction. This change in the direction and effective area of the voltage drop provides compelling evidence that liquid metal can effectively modulate the electrical pathways on the platinum-coated TPU fibre mat electrode.

The extracted SEM image was processed using commercial image analysis and processing software for contour extraction and vectorisation, as indicated by the green curve in Fig. 2c. The vectorised electrode contours were imported into COMSOL and simplified to 2D electrodes. Based on the aforementioned analysis, a voltage (*U*_0_ = 0.6 mV) was applied along both sides of the X-axis and Y-axis directions to simulate the potential drop of the electrodes before and after the increase of the edge-locking liquid metal. The resulting current density contour maps are presented in Fig. 2f, g. These current density maps reveal that the original platinum-plated TPU fibre mat electrode possesses a solitary conductive pathway and is on the brink of electrical failure due to localised cracking, which results in an extremely constricted current channel. With the addition of edge-locking liquid metal, the conductive pathways become more numerous and intricate, allowing the current to be transmitted in parallel through multiple interwoven electrical channels. Even if the current channels are constricted in some localised areas due to cracking, the current remains stable thanks to the redundant pathways. This stability arises from the electrical disruption caused by the multitude of Pt fractures along the fibres in the X direction due to external tensile strains, while the number of Pt fractures along the fibres in the Y direction remains minimal. The addition of the edge-locking liquid metal redirects the current transfer from the X-axis to the Y-axis. This shift enables the platinum-plated TPU fibre mat electrodes with the liquid metal at the locking edge to endure greater strain. This finding elucidates the edge-locking liquid metal’s capability to effectively modulate the response of the platinum-plated TPU fibre mat electrode. Using the same methodology, we also examined platinum electrode cracks on TPU films (refer to Note S2 and Fig. S7b-d). The results indicate that while the platinum-plated TPU film electrodes with the addition of edge-locking liquid metal can tolerate large strains, they are not suitable for sensor applications due to their minimal response to strain, a conclusion that aligns with the findings in Section “Ultra-high sensitivity, large-range fabric strain sensors”.

The investigation into the resistive behaviour of the proposed strain sensor is crucial for understanding its functionality and optimising its design. The transformation of the electrical pathways in the platinum-plated TPU fibre mats under tensile strain, particularly when modulated by edge-locking liquid metal, is schematically represented in Fig. 2h, i. These illustrations highlight a significant change in the sensor’s electrical performance. When the sensor is subjected to X-axis tensile strain, the conductive fibres aligned with the X-axis direction are the first to electrically fail, as indicated by the grey colour. In contrast, the conductive fibres oriented towards the Y-axis direction remain conductive, shown in blue, and are capable of enduring greater tensile strains. Initially, the electrical pathways are predominantly along the X-axis, which results in rapid failure of the electrodes as the stretching proceeds. However, with the introduction of liquid metal into the edge-locking structure, the primary electrical pathways shift towards the Y-axis, slowing down the rate of electrode failure. To elucidate this resistivity behaviour, models have been developed to describe the resistance characteristics separately, as depicted in Fig. S8a, b. The resistance of the platinum-plated TPU fibre mat electrodes before and after the addition of the edge-locking liquid metal is represented by the following:

1 $$ R = \frac{{\left( {R_{v} + R_{t} } \right)R_{o} }}{{R_{v} + R_{t} + R_{o} }} = \frac{{R_{o} }}{{1 + {\raise0.7ex\hbox{${R_{o} }$} \!\mathord{\left/ {\vphantom {{R_{o} } {R_{v} + R_{t} }}}\right.\kern-0pt} \!\lower0.7ex\hbox{${R_{v} + R_{t} }$}}}} $$

2 $$ R = \frac{{R_{v} }}{{1 + {\raise0.7ex\hbox{${R_{v} }$} \!\mathord{\left/ {\vphantom {{R_{v} } {R_{t} }}}\right.\kern-0pt} \!\lower0.7ex\hbox{${R_{t} }$}} + {\raise0.7ex\hbox{${R_{v} }$} \!\mathord{\left/ {\vphantom {{R_{v} } {R_{o} }}}\right.\kern-0pt} \!\lower0.7ex\hbox{${R_{o} }$}}}} $$

Here, *R* denotes the total resistance of the sensor, while *R*_*t*_, *R*_*v*_, *R*_*o*_ correspond to the resistance contributions from the platinum layer on the fibres along the stretching direction, the vertical direction of the stretching direction and the inclined direction, respectively. During the stretching process, the platinum layer on the fibres distributed along the stretching direction (X-axis) is the first to break, turning *R*_t_ into an open circuit. Subsequently, as the strain increases, the platinum layer on the inclined fibres fractures, causing *R*_*o*_ to become an open circuit. According to Eq. (1), when* R*_*o*_, the platinum-plated TPU fibre mat electrode fails before the addition of edge-locking liquid metal. However, after the integration of the edge-locking liquid metal, even as the stretching continues, the Pt layer on the fibres perpendicular to the stretching direction does not break. As per Eq. (2), when *R* = *R*_*v*_, the platinum-plated TPU fibre mat electrode remains electrically conductive, indicating an increase in the failure strain of the electrode. This demonstrates that the edge-locking liquid metal can effectively modulate the response of the platinum-plated TPU fibre mat electrode, enhancing its durability and functionality.

Furthermore, Fig. S9a illustrates that numerous cracks form in the platinum on the fibres under tension, causing microscopic electrical damage and a macroscopic increase in resistance. However, due to the confinement provided by the attached TPU fibres, the cracked Pt can reattach when the tensile strain is released, resulting in a decrease in resistance and recovery of the sensor’s functionality. SEM images presented in Fig. S9b, c, captured at random locations on the platinum-plated TPU fibre mats during and after the application of tensile strain (*ε*_stretch_ = 1), confirm that the platinum cracks rejoin upon strain release. This observation indicates that the strain sensor exhibits repeatable and reliable performance, making it suitable for applications requiring cyclic strain measurements.

### Response Regulation of Fabric Strain Sensor

The sensitivity and range of piezoresistive strain sensors are critical parameters that often exhibit a trade-off relationship: as sensitivity increases, the measurable range tends to decrease. To fully harness the sensor’s sensitivity, it is essential to modulate the sensor’s range to align precisely with the measurement requirements. The use of edge-locking liquid metals for modulating the response of platinum-plated TPU fibre mat electrodes has proven to be an effective electrical modulation technique.

**Fig. 3:**
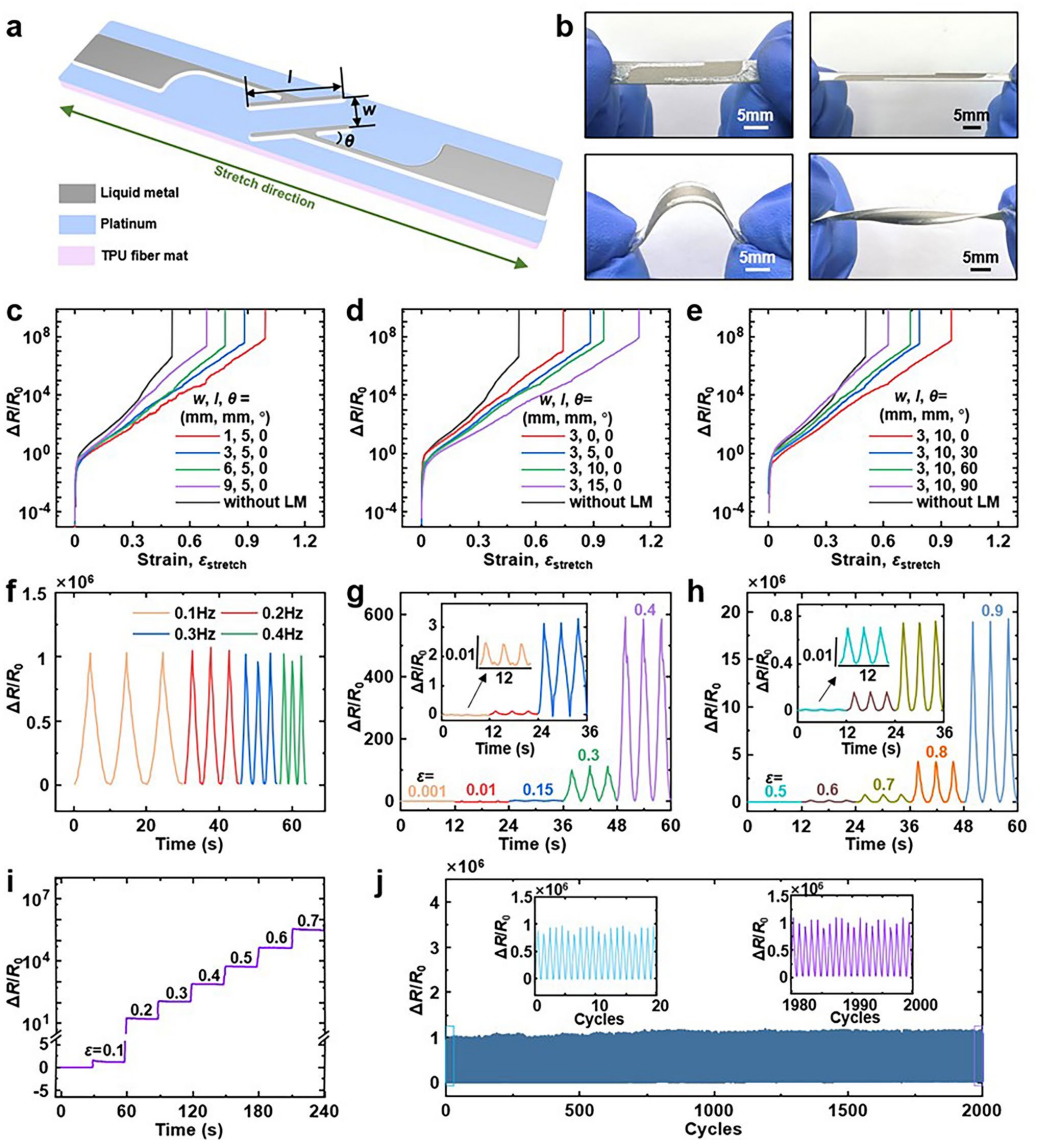
Response behaviour of platinum-plated TPU fibre mat fabric strain sensors coated with edge-locking liquid metal. **a** Schematic 3D structure of the sensor and geometrical feature dimensions of the patterned liquid metal. Overlap length: *l*, spacing: *w*, angle: *θ*. **b** Optical images for the fabricated strain sensors at the original, stretched, bent, and twisted states. Geometrical characteristic dimensions of patterned liquid metal in the sensor: *l* = 10 mm, *w* = 3 mm, *θ* = 0°. **c**–**e** are the response tests of the strain sensors obtained by varying the geometrical dimensions of the patterned liquid metal with spacing *w*, overlapping length *l* and angle *θ*, respectively. The black curves are shown as the control group without edge-locking liquid metal. **f** Dynamic stability of the strain sensors at various frequencies ranging from 0.1 to 0.4 Hz. Dynamic stability of the strain sensors at **g** small strains ranging from 0.001 to 0.4 and **h** large strains ranging from 0.5 to 0.9. The insets in **g** and **h** are magnified details. **i** Quasi-static response of the sensor with the strain increases by 0.1 for every 30 s, from 0 to 0.7. **j** Long-time electrical responses under 0.7 cyclic strain, with a frequency of 0.25 Hz for 2000 cycles

Systematic investigation of the geometrical characteristics of the patterned liquid metal is crucial for on-demand modulation of the sensor response. As described in the locking edge structure pattern, the geometric features of the patterned liquid metal are categorised into overlap distance (*w*), overlap length (*l*), and angle (*θ*), as depicted in Fig. 3a. The liquid metal is coated onto the platinum-plated TPU fibre mat using a laser-machined skeletonised stainless steel mesh as a mask, facilitating the creation of strain sensors. The superior flexibility of the sensor is evident in the optical photographs shown in Fig. 3b, which capture the sensor in its initial, tensile, bending, and torsion states.

Following the fabrication of strain sensors with varying geometries, their performance is tested and analysed using the control variable method. Figure 3c–e presents the electromechanical response test results for strain sensors with varying overlap distances (*w*), overlap lengths (*l*) and clamp angles (*θ*), with the black curves representing control samples. The sensor’s failure strain *ε*_max_ exhibits different relationships with these geometric parameters:

- Variable overlap distance *w*: Test results for overlap distances of 1, 3, 6 and 9 mm are displayed in Fig. 3c, with a fixed overlap length (*l*) of 5 mm and an angle (*θ*) of 0°. Figure S10a shows the variation of sensor failure strain *ε*_max_ with respect to overlap distance *w* for this case: $\varepsilon_{\max } = 1.012 - 0.037{\text{mm}}^{ - 1} w\;(R^{2} = 0.9853)$.
- Variable overlap length *l*: Test results for overlap lengths of 0, 5, 10 and 15 mm are shown in Fig. 3d, with a constant overlap distance (*w*) of 3 mm and an angle (*θ*) of 0°. Figure S10b shows the variation of sensor failure strain *ε*_max_ with overlap length *l* for this case: $\varepsilon_{\max } = 0.740 + 0.025{\text{mm}}^{ - 1} l$ $(R^{2} = 0.9754)$.
- Variable pinch angle *θ*: Test results for overlap lengths of 0, 5, 10 and 15 mm are shown in Fig. 3e, with a constant overlap distance (*w*) of 3 mm and an angle (*θ*) of 0°. Figure S10c shows the variation of sensor failure strain *ε*_max_ with clamp angle *θ* for this case: $\varepsilon_{\max } = 0.932 - 0.004^{ - 1} \theta$ $(R^{2} = 0.9588)$.

The strain sensor’s range decreases with an increase in overlap spacing (*w*), increases with an increase in overlap length (*l*) and decreases with an increase in clamp angle (*θ*). Utilising these relationships, the sensor’s response can be tailored and designed to meet specific requirements.

It is important to note that the test graphs’ vertical coordinates are logarised, and the processed curves exhibit an almost linear increase in the effective section. This indicates that the rate of change of the sensor’s resistance to strain follows an exponential response function. Figures S11-S13 provide graphs of the curves without logarisation of the longitudinal coordinates and the corresponding fitted curves for each. The chosen exponential fitting function is represented as: $\frac{\Delta R}{R} = a({\text{e}}^{{b\varepsilon_{{{\text{stretch}}}} }} - 1)$. The coefficients *a* and *b* correspond to different geometrical parameters of the liquid metal, and the fit R^2^ values are displayed in Table S1, with all fits exceeding 0.999. The slopes of the fitting equations, i.e. the Gauge Factor, can be determined as:

3 $$ Gauge\;Factor = abe^{{b\varepsilon_{stretch} }} $$

According to Eq. (3), the Gauge Factor is also an exponential function with respect to strain. This exponential form endows the sensor with the capability to modulate its sensitivity by pre-stretching during operation. It is crucial to highlight that the proposed sensor can modulate its response not only through geometrical alterations in the liquid metal patterning but also by a secondary modulation through pre-stretching. The range and sensitivity of the proposed sensor surpass those of existing crack and liquid metal-based strain sensors in the wearable range (50%–100%), with a Gauge Factor reaching 10^8^ or higher and a range extending to 100% or more, as compared in Fig. 1f. The detailed statistics of the range and sensitivity of sensors from various literature are provided in Table S2.

To further explore the sensor’s response, liquid metal geometrical feature sizes with an overlap distance (*w*) of 3 mm, an overlap length (*l*) of 10 mm and a pinch angle (*θ*) of 0° were selected as typical parameters. This typical parameters are chosen to take into account a maximum strain value of about 0.9 in human skin [5]. The real-time rate of change of the sensor’s resistance was recorded to assess its performance, such as electrical stability, in detecting diverse strains. Figure 3f presents cyclic tensile tests at different frequencies (from 0.1 to 0.4 Hz) at an applied strain of 0.7, demonstrating excellent dynamic stability. The sensor is sensitive and highly reproducible to both small strains (range 0.001–0.4, as shown in Fig. 3g) and large strains (range 0.5–0.9, as shown in Fig. 3h), with the inset displaying an enlarged signal that appears to have a negligible response. The electrical stability of the sensor under staged static strains (from 0 to 0.7) is shown in Fig. 3i, with a strain increment of 0.1 every 30 s. Remarkably, the proposed fabric strain sensor exhibits exceptional mechanical and electrical stability even under large strains, as depicted in Fig. 3j, where the sensor can endure 2000 cyclic tensile tests at a frequency of 0.25 Hz and a strain of 0.7, with only minor fluctuations and rises in the response. The primary cause of signal drift is the mechanical fatigue of the rubber, an inherent material property. Altering the material system could potentially enhance signal stability. To ensure long-term sensor stability, a two-week test on the rate of change of resistance was conducted, as shown in Fig. S14. The results confirm that the sensor’s performance remains stable over this extended period. Furthermore, the sensor has good cycling mechanical properties as shown in Fig. S15. The temperature response of the sensor was tested as shown in Fig. S16, and the sensor responded weakly to changes in temperature (resistive temperature coefficient as low as 6.494 × 10^–4^). For ultra-high sensitivity sensors, the amplitude of this weak response signal due to temperature change will be negligible in the strain response signal curve. In addition, in epidermal electronics application scenarios where the temperature of the human skin surface is within a narrow range, this stable temperature environment will further weaken the temperature response signal of the sensor.

### Vapour Permeability

**Fig. 4:**
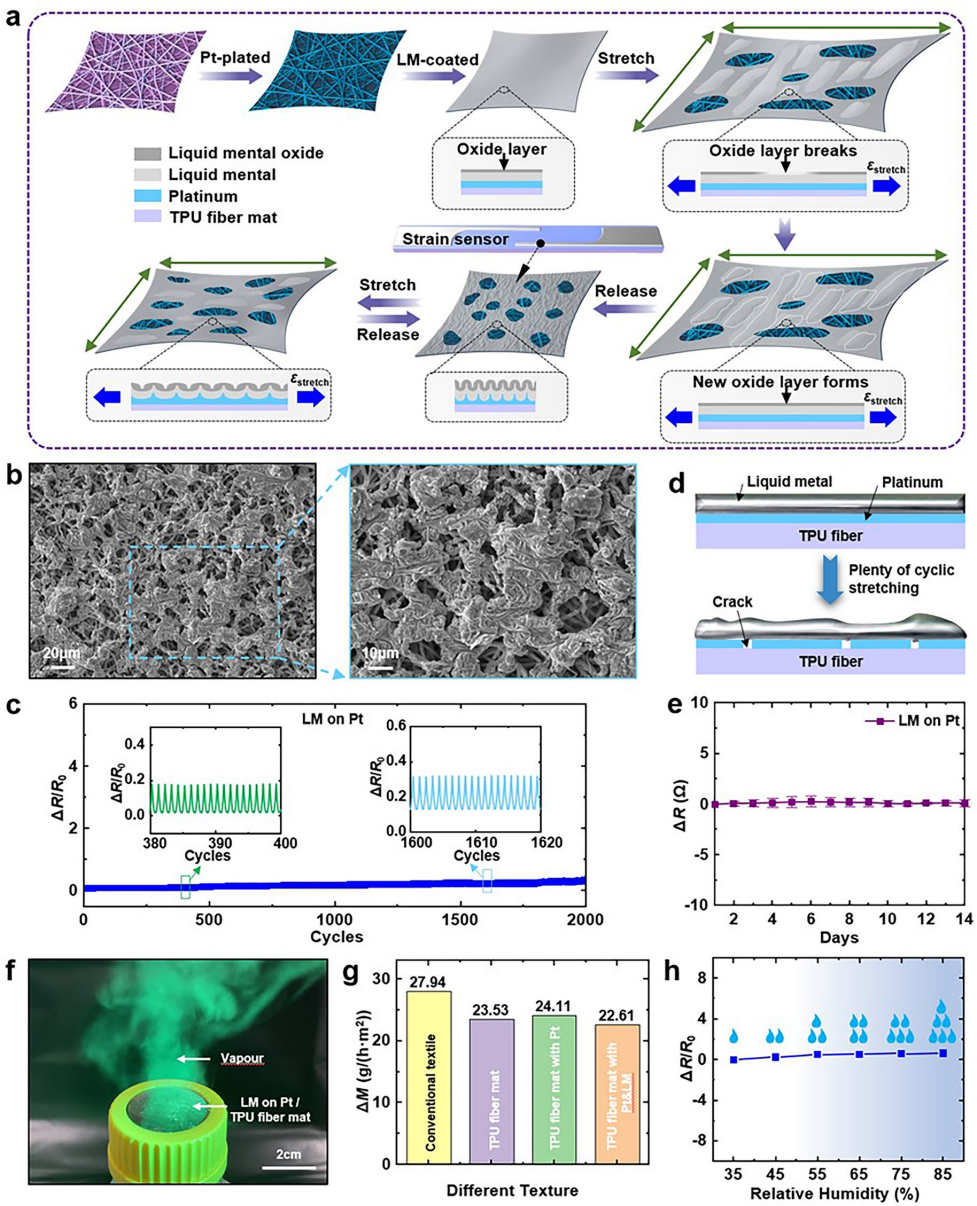
Stability and permeability of the edge-locking liquid metal. **a** Schematic diagram of the process for making liquid metal breathable. **b** SEM images of liquid metals with porous structures which remain electrically connected. **c** The long-term electrical response of platinum-plated TPU fibre mats coated with liquid metal under 100% cyclic strain at 0.25 Hz for 2000 cycles. **d** Schematic diagram of liquid metal remaining connected after a large number of cycles of stretching following the coating of liquid metal on a platinum-plated TPU fibre mat. **e** Long-term electrical stability of platinum-plated TPU mats coated with liquid metal. **f** Optical photograph of water vapour transmission through platinum-plated TPU mats coated with liquid metal. **g** Permeability of conventional cloths, TPU fibre mats, platinum-plated TPU fibre mats and liquid metal coated platinum-plated TPU fibre mats at a temperature of 25 °C and a humidity of 50%. **h** The resistance change ratio of the strain sensors versus relative humidity

Epidermal sensors are designed to be worn on the skin for extended periods, and as such, they must exhibit excellent biocompatibility to ensure comfort and prevent any adverse reactions such as itching or irritation. The skin naturally secretes sweat, and it is crucial for the sensor to either allow sweat to pass through or be resistant to the effects of sweat to maintain these properties. The breathability and moisture permeability of fabric sensors are particularly beneficial in this regard. Both pure and platinum-plated TPU fibre mats are known for their good air permeability, which is essential for comfortable wear. To ensure that the liquid metal region of the sensor remains breathable while maintaining stable electrical conductivity, a specific process method has been developed, as outlined in Fig. 4a. This method involves platinum coating the TPU fibre mats (via magnetron sputtering at 50 nm), followed by the application of liquid metal, and concludes with cyclic stretching of the fibre mats until the liquid metal’s colour darkens gradually (under *ε*_stretch_ = 1.5, 100 cycles). The resulting porous structure of the liquid metal on the TPU fibre mats, as shown in Fig. 4b, confirms the effectiveness of this approach. A systematic study will be conducted to evaluate the electrical stability and permeability of the liquid metal electrodes.

The 0–100% cyclic strain loading test on the platinum-plated TPU fibre mats coated with liquid metal, as shown in Fig. 4c, demonstrates that during up to 2,000 cyclic loading cycles, the signals are well reproducible without any perturbation or electrical failure. This indicates that the electrodes of the liquid–metal coated platinum-plated TPU fibre mat electrodes possess excellent electrical stability. There is virtually no change in the pore scale of the liquid metal as can be seen in the SEM image after cyclic stretching (Fig. S17), which again illustrates the stability of the electrode performance of the platinum-plated TPU fibre mats coated with the liquid metal. To emphasise the significance and necessity of the platinum metal layer, a 0–100% cyclic strain loading test was also performed on the liquid metal coated but not platinum-plated TPU fibre mats, as depicted in Fig. S18. The results show that the signal reproducibility of the liquid metal electrodes without platinum plating is poor, with breakage occurring quickly after more than a thousand cycles. This suggests that the electrical stability of the liquid metal electrode without the platinum metal cannot be guaranteed. Figure S19a displays an SEM image of the liquid metal on the TPU fibre mat after breakage, revealing a large amount of agglomeration of the liquid metal, leading to numerous interruptions between the liquid metals, which ultimately results in electrical failure. The schematic diagram of the agglomeration process occurring in the liquid metal is provided in Fig. S19b. In contrast, no agglomeration is observed when platinum is deposited on the TPU fibre mats first, followed by the application of liquid metal, as shown in Fig. 4d. The inherent property of fusion between liquid metal and platinum to form an alloy leads to a reactive wetting process of the coated liquid metal on the surface of the platinised TPU fibre mats [56]. Therefore, the occurrence of agglomeration can be avoided. It is worth mentioning that Zheng’s group has successfully achieved gas-permeable electrodes with high tensile strength by applying liquid metal directly on the surface of SBS fibre mats [57]. However, the liquid metal coated directly on the TPU fibre mats failed electrically after large tensile cycles due to agglomeration, which could be attributed to the difference in interfacial wettability due to the change of material system. The developed process effectively solved the above problem.

Figure 4e shows the resistance change test of the liquid metal-coated platinum-plated TPU fibre mat electrode for two consecutive weeks, and it can be seen that there is almost no change. Therefore, the electrode’s electrical dynamic and static stability are quite excellent. Building upon the established electrical stability of the liquid metal electrodes, tests were conducted to evaluate their ultimate tensile capabilities. As depicted in Fig. S20, the results from the tensile process show simultaneous measurements of resistance change and stress values alongside the strain. The inset in the figure provides an optical photograph of the sample during the test, visually confirming the stretchability of the electrode. The findings indicate that the liquid metal electrodes can be stretched to 400% or more without electrical failure. The ultimate electrical failure occurs only when the TPU fibre mat substrate undergoes complete fracture. Notably, even under such high tensile strain, the change in electrode resistance remains minimal, with an increase of less than 16 Ω. This small resistance change is insignificant for piezoresistive strain sensors with ultra-high sensitivity, ensuring that the measurement accuracy is not compromised. Furthermore, the stress–strain curves reveal that the Young’s modulus of the liquid metal electrode is approximately 2 MPa, which is comparable to the range of 0.5–1.95 MPa for human skin [58]. This similarity in mechanical properties suggests that the liquid metal electrodes can provide a comfortable wearing experience, closely matching the flexibility and elasticity of the skin. Figure S21a, b presents a schematic and an optical photograph, respectively, of an LED matrix powered by the liquid metal electrode acting as a section of wire. During the test, the strain *ε*_stretch_ on the liquid metal electrode was incrementally increased from 0 to 2 at 0.5 intervals while maintaining a constant voltage power supply. Despite the stretching, all LED matrices remained illuminated stably, without any significant dimming or flickering, further demonstrating the superior stretching ability and electrical stability of the liquid metal electrodes.

These results highlight the potential of liquid metal electrodes for applications in wearable electronics, where both mechanical flexibility and electrical reliability are crucial. The combination of high stretchability, low Young’s modulus and stable electrical performance positions these electrodes as strong candidates for next-generation wearable sensors and other conformable electronic devices.

Optical photographs of water vapour passing through the liquid metal electrodes visually demonstrate the excellent breathability (as shown in Fig. 4f). Figure 4g shows the average water vapour transmission rate ($\dot{m}$) of various materials (e.g. conventional textiles, TPU fibre mats, platinised TPU fibre mats and platinised TPU fibre mats coated with liquid metal) for one week at constant temperature (25 °C) and humidity (50%). The fabric-structured materials showed permeabilities ($\dot{m}$ = 22.61 to 24.11 g h^−1^ m^2^) close to those of conventional textiles ($\dot{m}$ = 27.94 g h^−1^ m^2^). Platinum plating on the upper surface or liquid metal coating after platinum plating has little effect on the permeability and ensures that the sensors are comfortable to wear. Figure 4h shows that the effect of humidity on the sensor resistance is negligible, suggesting that the proposed fabric sensors are electrically stable even when operating in sweaty environments. To simulate a sweat-like humid environment, a humidifier was used in which a 0.9% sodium chloride (NaCl) solution was employed. The TPU fibres are coated with a dense platinum film through the process of magnetron sputtering deposition, which ensures a uniform and high-quality layer with excellent electrical conductivity and stability. This platinum film is crucial for the sensor’s functionality, as it provides a conductive base that can reliably detect strain. The addition of liquid metal to the sensor design leverages its inherent properties of high ductility and consistent electrical conductivity, further enhancing the sensor’s performance. These materials, both the platinum film and the liquid metal, maintain their electrical properties across various humidity levels, ensuring the strain sensors remain stable and functional in different environmental conditions.

### Application Demonstrations

**Fig. 5:**
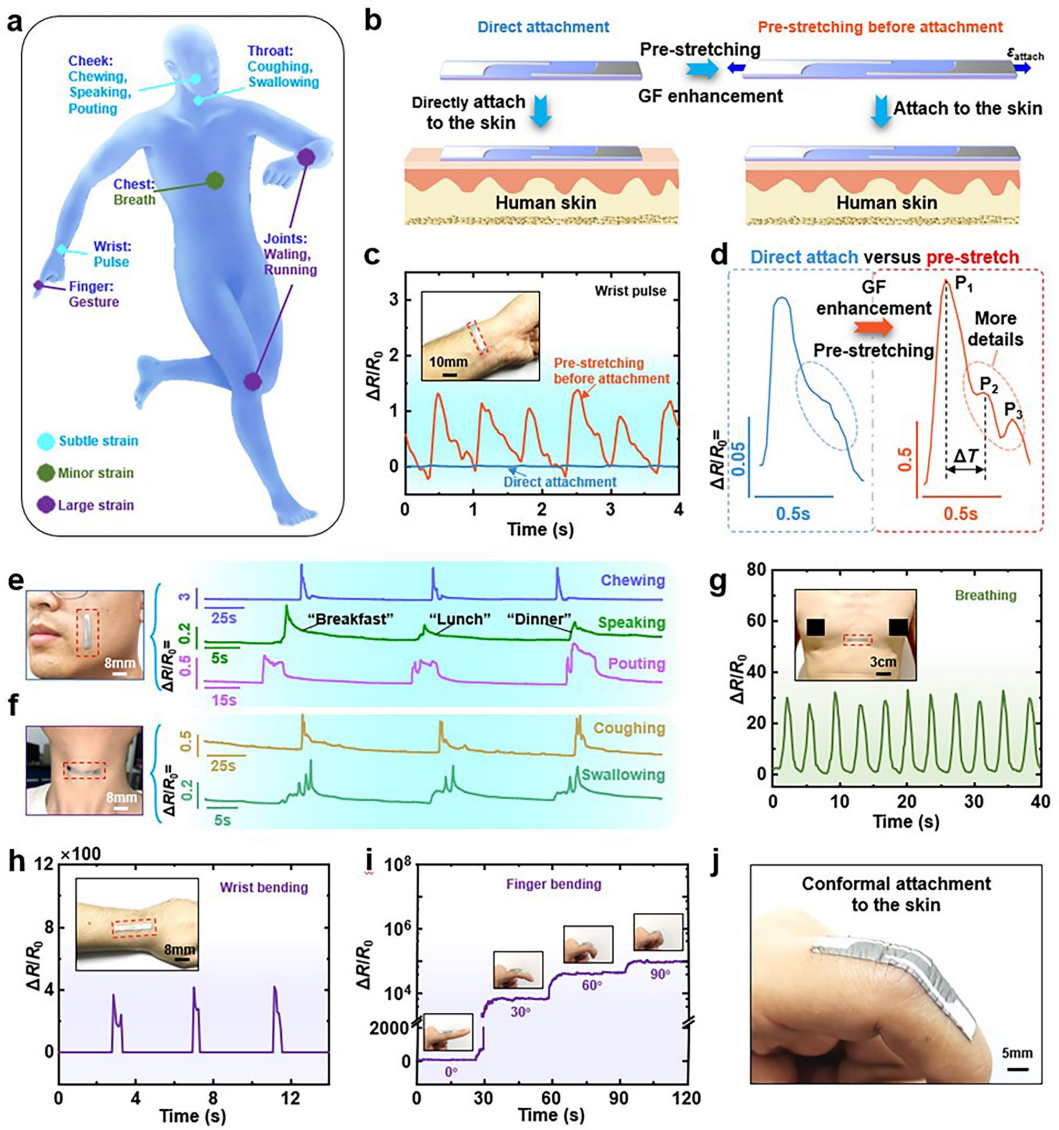
Demonstration of whole-body motion signal detection from subtle to large strains. **a** Schematic representation of motion signals that can be detected in different parts of the human body. **b** Schematic diagrams of the process of attaching strain sensors directly and after pre-stretching to the skin surface. **c** Comparison of pulse signals detected by a strain sensor attached to a human wrist before and after pre-stretching. Inset is an optical photograph of the strain sensor attached to the wrist. **d** The signals in **c** are magnified and compared. **e** Strain sensor is attached to the face to detect signals during chewing, articulation and grimacing. **f** Strain sensor is attached to the outer surface of the throat to detect signals during coughing and swallowing. **g** Strain sensor is attached to the chest for respiratory signal detection. **h** Strain sensor attached to the back of the wrist for signal detection of wrist bending. **i** The resistance change ratio of the strain sensor on a finger at different bending angles 0°, 30°, 60° and 90°, respectively. **j** Optical image of a strain sensor on a finger

The human skin’s surface experiences a variety of strains that can offer valuable insights into physiological conditions. The precise detection of these epidermal strains is advantageous for applications such as health monitoring and movement analysis. These strains vary in intensity, ranging from subtle vibrational strains like pulses, to muscle strains associated with facial expressions, to more pronounced strains from joint movements and gestures, as depicted in Fig. 5a. The fabric strain sensor introduced in this context is designed to offer a balance between ultra-high sensitivity and a large volume range. This dual capability allows the sensor to effectively detect the entire spectrum of strain scenarios, from the most delicate to the most substantial. The previous analyses have demonstrated the ability of the proposed sensor to measure weak signals. In order to achieve a more accurate perception of weak strain signals, we propose here an additional method that can increase the sensitivity of the sensor by pre-stretching the strain sensor during the pasting process. Figure 5b shows the schematic diagrams of direct and pre-stretching attachment. An attachment strain *ε*_attach_ is applied to the sensor before attaching the sensor to the skin, which is calculated according to the Gauge Factor formula obtained in Section “Response regulation of fabric strain sensor”: $Gauge \, Factor = ab{\text{e}}^{{b\varepsilon_{{{\text{stretch}}}} }}$. It can be seen that the strain *ε*_stretch_ changes from 0 to *ε*_attach_ at the initial stage. Then the value of the initial GF changes from *ab* to $ab{\text{e}}^{{b\varepsilon_{{{\text{attach}}}} }}$. This significantly increases the initial sensitivity. Note that the form of the exponential function means that a very small pre-strain needs to be applied in order to obtain a significant increase in sensitivity, which means that the range of the sensor is sacrificed to a very limited extent. As a result, the tendency of the sensor to return to its original state is very weak and does not affect the long-term reliability of the measurement. In the application scenario of wrist pulse measurement, the applied pre-strain is about 0.075, and the initial values of the sensor gauge factor before and after applying the pre-strain can be calculated as 4.534 and 20.693, respectively, according to the above formula, which shows that the weak pre-stretching led to a significant improvement. Here we only make a simple application demonstration, more application scenarios and requirements can be adjusted appropriately according to the above calculation process. And because the TPU fibre mats inherently have a small Young’s modulus, this means that the contraction force applied by the sensor is very weak and does not affect wearer comfort. The pulse signal measurements before and after applying the adhering strain *ε*_stretch_ are shown as blue and orange curves in Fig. 5c, respectively, and the inset is an optical photograph of the sensor attached to the wrist. The amplitude of the pulse signal measured after applying the adhering strain is increased by about ten times, which is the result of the increase in sensitivity. In addition, the pulse signal obtained after the increase in sensitivity has more detailed information. As shown in Fig. 5d, the waveforms of single-cycle signals before and after sensitivity enhancement are shown in the blue and red curves, with three typical peaks in the red curve consistent with the standard pulse waveform and only one typical peak in the blue curve. The early systolic peak (P1) resulted from blood squeezing out of the heart, followed by an inflection point (P2), and the dicrotic notch and dicrotic peak (P3) resulted when blood flowed from the heart during aortic valve closure [59]. By recording three typical peaks in a single pulse, potential health issues of the human body could be effectively distinguished. The time interval between the systolic (*T*_P1_) and inflection point (*T*_P2_) was defined as the digital volume pulse (Δ*T*). Information such as pulse period, amplitude of different waves and ΔT could be used to determine the heart rate, atherosclerosis status and other health states. In summary, the sensitivity of the sensor can be effectively enhanced by elevating the applied pre-strain without significant performance sacrifices or wearer comfort degradation. Note that the method of increasing the sensitivity by pre-stretching is an additional means to further enhance the sensitivity of the sensor and is only demonstrated in the pulse measurement scenario. Due to the inherent high sensitivity of the sensor, the subsequent scenarios are shown with direct attachment.

The fabric strain sensors, with their exceptional sensitivity and wide-ranging detection capabilities, are highly adaptable for a variety of physiological monitoring tasks. When applied to the face, these sensors can accurately detect the subtle strains of facial muscles during activities such as chewing, speaking and expressing emotions, as shown in Fig. 5e. This precision is invaluable for interpreting silent speech and facial expressions, offering a critical communication aid for individuals who have lost their voice. On the neck, the fabric strain sensors can effectively gauge muscle strains related to coughing and swallowing, as depicted in Fig. 5f. Identifying these strain patterns can be essential in the early identification and ongoing monitoring of conditions like dysphagia or respiratory infections. For respiratory function assessment, the sensors can be positioned on the chest, as illustrated in Fig. 5g. The data collected from the chest contour provides insights into the state of the respiratory system. This type of monitoring is beneficial not only for athletes undergoing respiratory training but also for tracking and managing sleep apnoea and other respiratory disorders. Additionally, by attaching the fabric strain sensors to the wrist, knuckle and knee joints, they can monitor limb movements, as shown in Figs. 5h, i and S22. The sensors can detect changes in joint angles, enabling motion monitoring and sign language recognition, which is immensely valuable for assistive technologies serving the deaf and hard-of-hearing community. The versatility of these fabric strain sensors spans numerous applications, from health monitoring and disease management to improving communication and enhancing assistive technologies.

It is worth mentioning that the sensors are incredibly thin, with a thickness of less than 50 μm, which enables them to be conformally adhered to the skin surface, as shown in the optical photograph in Fig. 5j. This conformal attachment is facilitated by van der Waals forces, which ensure a strong yet non-invasive bond. By minimising the space between the sensor and the skin, this method reduces the output of extraneous signals, thereby enhancing the accuracy of strain data acquisition. To achieve accurate strain detection, the sensor must possess a low Young’s modulus and be as thin as possible. A low Young’s modulus allows the sensor to closely follow the skin’s movements, while thinness ensures minimal interference with the skin’s natural flexibility. The inherent flexibility of fabric sensors is a natural advantage in this regard. The thickness of the sensor is primarily determined by the TPU fibre mat, which can be manipulated during the electrospinning process to achieve ultra-thin fibre mats. By controlling the electrospinning time, it is possible to produce fibre mats with the desired level of thinness, which in turn allows for the creation of ultra-thin, highly sensitive fabric strain sensors. In summary, the proposed fabric strain sensor combines high sensitivity with a broad detection range, conformal adhesion to the skin and minimal thickness to provide precise and reliable monitoring of a wide range of physiological strains. Their conformal, lightweight design, combined with high sensitivity and reliability, renders them ideal for wearable technologies that can discreetly monitor human physiological signals without disrupting daily activities. It should be noted that in practice the generation of too many extraneous signals (e.g. pressure signals) should be avoided as far as possible. The potential for these sensors to impact various facets of healthcare and daily life is significant and promising.

## Conclusions

In conclusion, we have developed a stretchable piezoresistive fabric strain sensor based on platinum cracks on elastic fibre mats, which provides extremely high sensitivity with a large range. On-demand tuning of the sensor response is achieved by modifying the strain-sensitive electrical pathways through patterning of the liquid metal, and not only that, a second tuning of the sensor response can be achieved by applying applied strain during use. Detailed mechanical and electrical analyses elucidate the underlying mechanisms and the influence of key factors, which can effectively provide guidance for the modulation of the stretchable electrode response by electrical methods. The microstructure of porous fabrics leads to good air permeability and low Young’s modulus for excellent wearing comfort. On this basis, we have solved the air permeability problem of liquid metal electrodes and achieved good electrical stability. Accurate measurements ranging from weak strains from pulse vibration to large strains from joint rotation demonstrate the sensor’s excellent performance and extraordinary application prospects.

## Supplementary Information

Below is the link to the electronic supplementary material.

Supplementary file1 (DOCX 4643 KB)
