# Supplementary material for: Flexible Strain Sensors with Ultra-High Sensitivity and Wide Range Enabled by Crack-Modulated Electrical Pathways
Source: Nanomicro Lett. 2024 Nov 18;17:64. doi: 10.1007/s40820-024-01571-6 (PMC11570575; doi:10.1007/s40820-024-01571-6)
Supplement: Supplementary file 1 — Supplementary file1 (DOCX 4643 KB) [file 40820_2024_1571_MOESM1_ESM.docx]

**Supplementary Note 1. Finite element analysis of TPU fibre networks**

In order to analyse the strain distribution of the TPU fibre network as a whole when subjected to tensile strain, finite element simulations were carried out using the commercial software ABAQUS. The intrinsic stress-strain characteristics of the TPU material were tested by tensile testing equipment and fitted using different hyperelastic models Ogden3, Neo-Hookean, Mooney-Rivlin1 and Yeoh as shown in Supplementary Figure 4, where the blue points are the stress-strain characteristics obtained from the tests. The Ogden3 model was used as the material model used for the simulations due to the minimal bias in the fitting of the hyperelastic model. Ideally, the disordered fibres are uniformly distributed along all directions. Therefore, the angularly homogeneous but randomly disordered fibres were used as the fibre network, and the finite element simulation results of applying tensile strain along the X-axis direction are shown in Supplementary Figure 5, where it can be seen that as the fibre network is subjected to a larger tensile strain, the axial tensile strain is greatest in fibres along the X-axis, and also first reaches the electrical failure strain of the platinum on the TPU film. Where the failure strain is 0.15, which is given based on the experiment. While the fibres along the y-axis direction are subjected to the least tensile strain.

**Supplementary Note 2. Electrical analysis of platinum cracks on TPU films**

Supplementary Figure 7a shows SEM images of platinum cracks in platinum-plated TPU film electrodes subjected to tensile strains of 0,0.1,0.3, and 0.5, respectively, and it can be seen that when the platinum-plated TPU film electrodes are subjected to tensile strains in the direction of the X-axis, penetrating-type straight cracks along the Y-axis direction are produced very quickly. As the tensile strain increases, the number of penetrating straight cracks becomes larger, but cracks along the tensile direction, which is the X-axis direction, rarely occur.

The region in the red dashed box of the SEM image of the platinum electrode crack on the TPU film with *ε*_stretch_ = 0.3 in Supplementary Figure 7a was extracted as shown in Supplementary Figure 7b. The extracted SEM image was processed by contour extraction and vectorisation using the commercial image analysis and processing software as shown by the green curve. The vectorised electrode contours were input into COMSOL and reduced to 2D electrodes. Based on the results of the above analysis, a voltage (*U*_0_=0.6mV) was applied along both sides of the X-axis direction and along both sides of the Y-axis direction to simulate the potential drop of the electrodes before and after the increase of the edge-locking liquid metal, and the obtained current density cloud diagrams are shown in Supplementary Figure 7c and d. It can be seen that the cracked electrode is electrically failed (current density of 0) before the addition of the edge-locking liquid metal, while after the addition of the edge-locking liquid metal, there is almost no area of electrical failure and high current density is transmitted in parallel along the Y-axis. This illustrates the fact that after the addition of the liquid metal of the locking edge structure, the platinum-plated TPU film electrodes, although capable of withstanding large strains, have a very small response to strain and are not suitable for use as sensors.


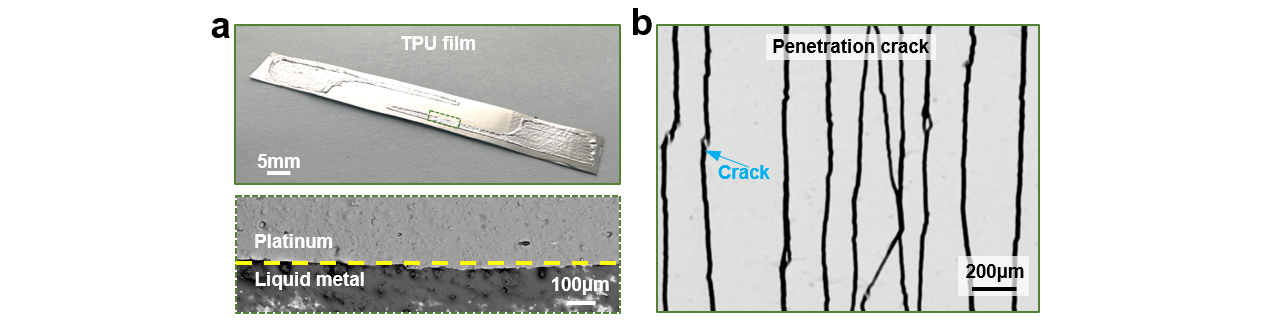


**Supplementary Figure 1. a** Optical image of a platinum-plated TPU film coated with edge-locking liquid metal on a release paper (top). SEM image of the edge of the patterned liquid metal locally enlarged (bottom). **b** SEM image of the platinum-plated TPU film at transverse strain *ε*_stretch_=0.3.


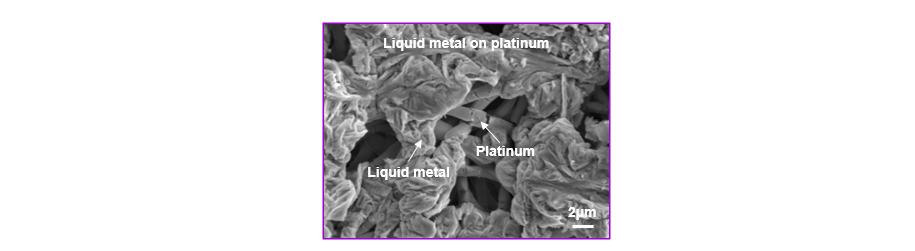


**Supplementary Figure 2.** Partial enlargement of the platinum-plated TPU fibre mat coated with liquid metal, which still has a porous and breathable structure.


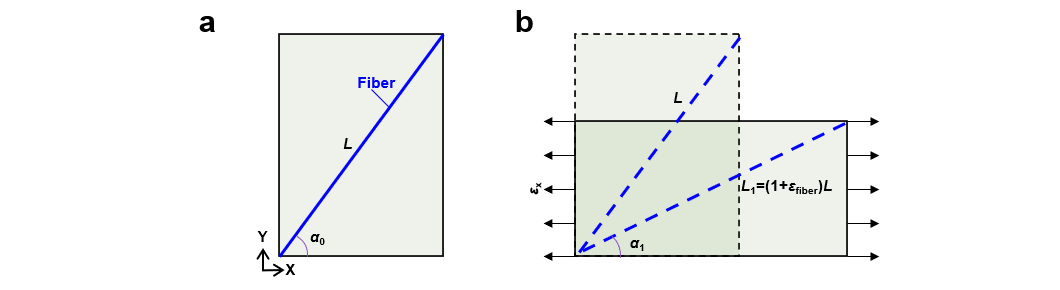


**Supplementary Figure 3.** Deformation relationship between fiber and substrate on the strain sensor before (**a**) and after (**b**) stretching.


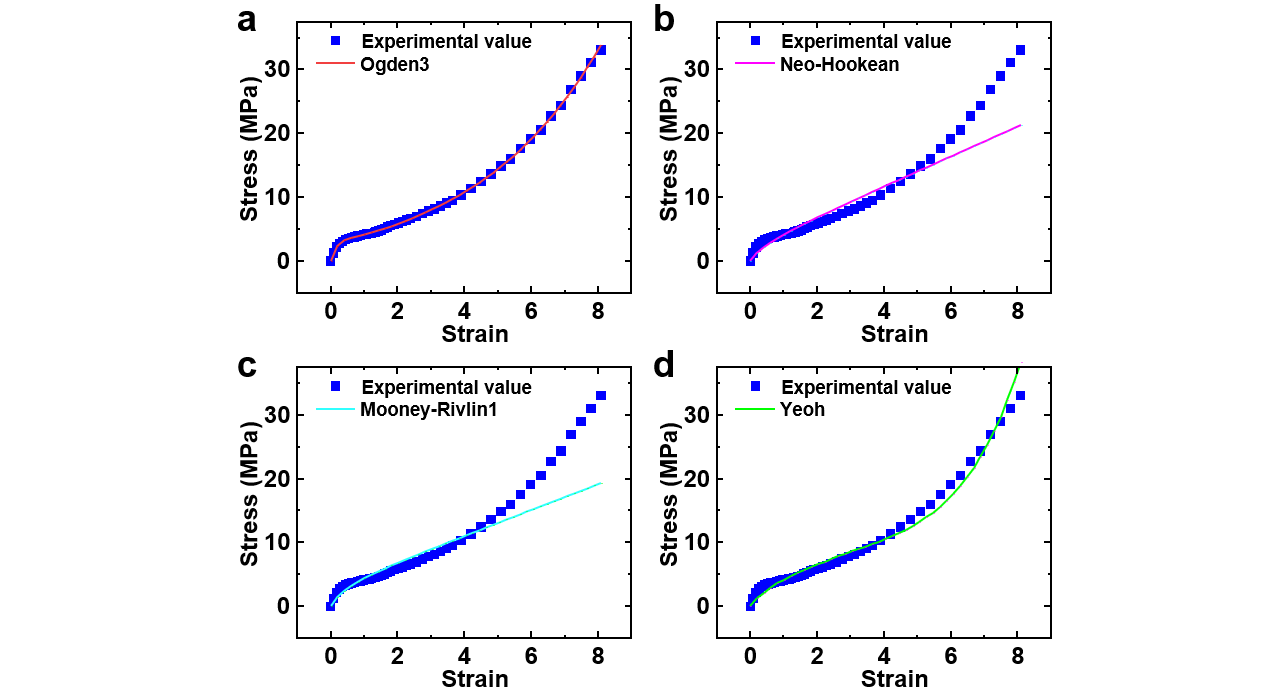


**Supplementary Figure 4. a**, **b**, **c** and **d** correspond to the results of fitting the Ogden 3, Neo-Hookean, Mooney-Rivlin1 and Yeoh hyperelasticity models and the experimental test data of the TPU film, respectively. Scattered points are the respective experimental data.


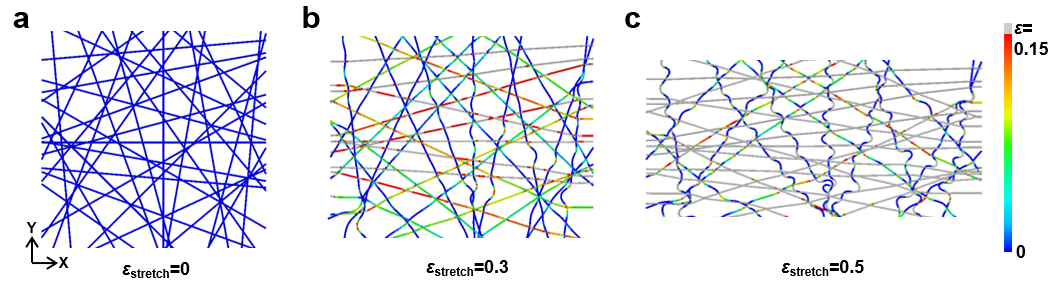


**Supplementary Figure 5. a** Randomly distributed TPU fibre network. **b** and **c** are strain field cloud plots when the whole is subjected to uniaxial strains of 0.3 and 0.5, with the grey portion being the region of strain > 0.15, i.e., the critical strain for electrical failure of platinum-plated TPUs during tensile testing.


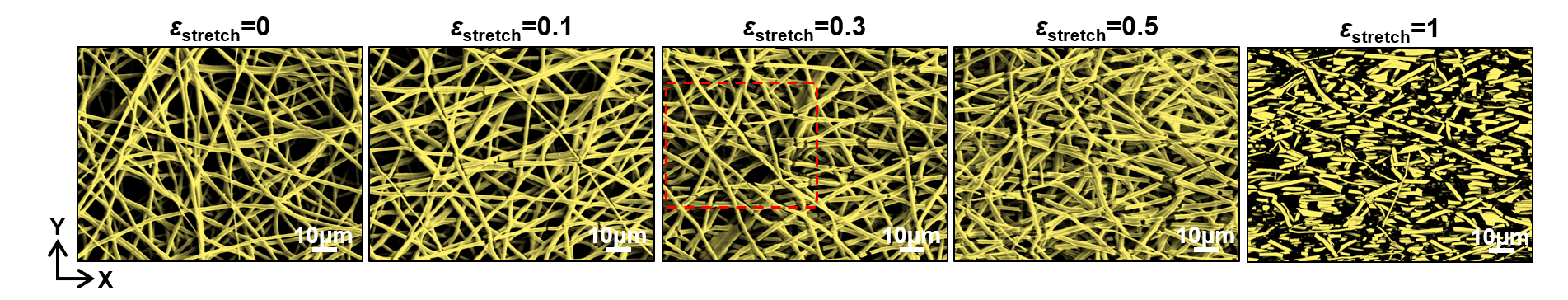


**Supplementary Figure 6.** SEM images comparison of platinum cracks on TPU fibre mats under different x-axial strain conditions. The strains are 0, 0.1, 0.3, 0.5 and 1.


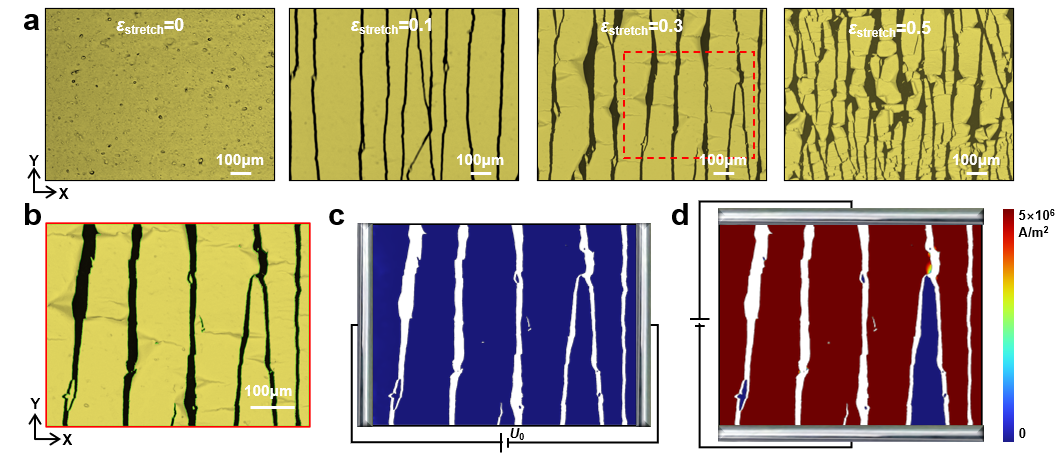


**Supplementary Figure 7. a** SEM comparison of platinum cracks on TPU films under different x-axial strain conditions. The strains are 0, 0.1, 0.3 and 0.5. **b** Magnification of the red dashed box part of the SEM image in **a** at the strain of 0.3. The green line in the figure shows the edge of the electrode. **c** and **d** are the current density cloud plots after applying the potential U_0_ along the X and Y directions, respectively.


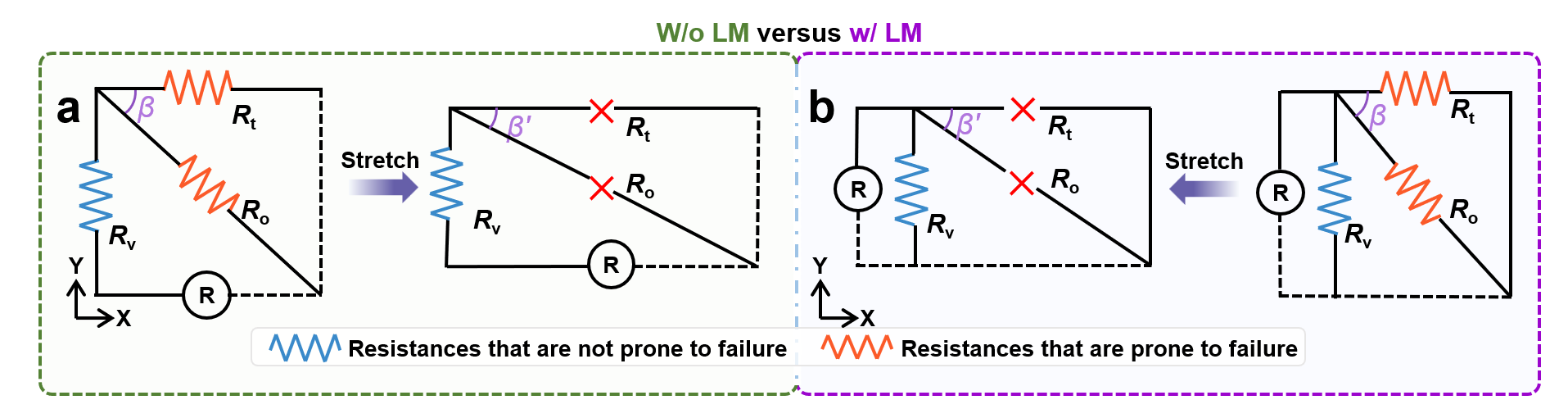


**Supplementary Figure 8. a** and **b** are schematic diagrams showing the resistance model of platinum-plated TPU fibre mats before and after the application of edge-locking liquid metal, respectively.


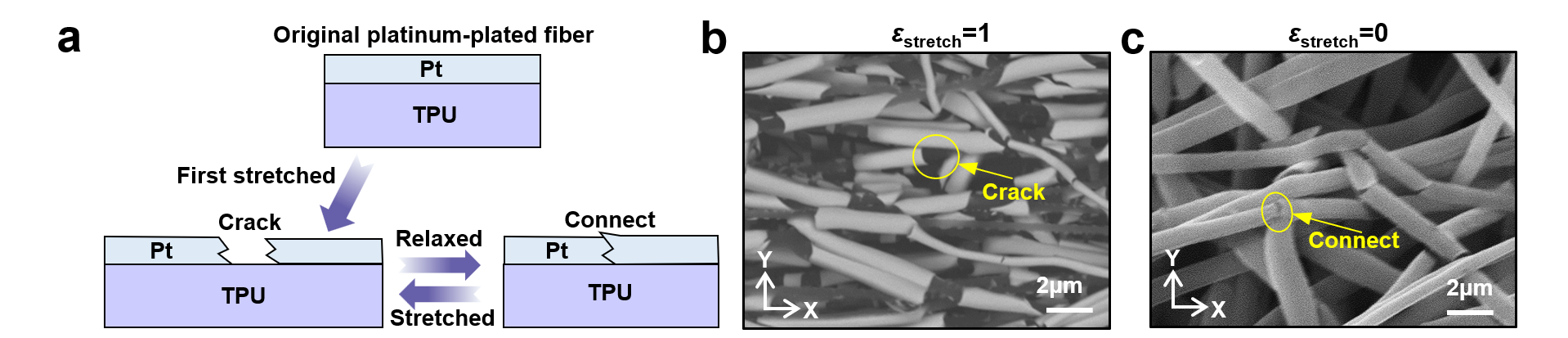


**Supplementary figure 9.** Schematic diagrams (**a**) and SEM images (**b** and **c**) of the platinum layer on the TPU fibres cracked and re-lapped under stretching and relaxation conditions.


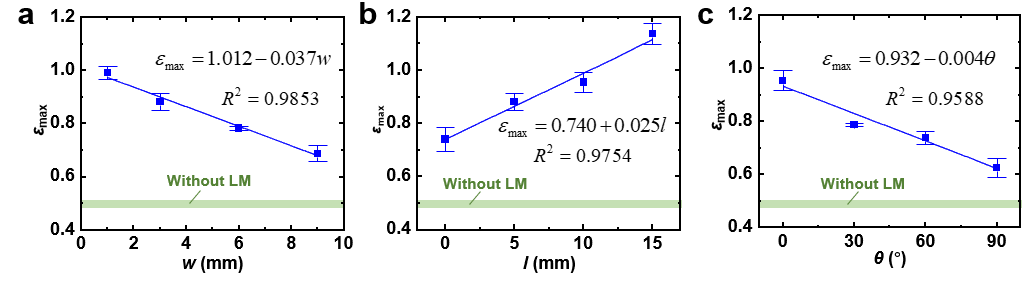


**Supplementary Figure 10. a** Fitted curves for the variation of electrical failure strain with overlap spacing (*w*) when the overlap length (*l*=5mm) and angle (*θ* = 0°) are kept constant. The green bar represents the electrical failure strain of the platinum-plated TPU fibre mats without edge-locking liquid metal. **b** Fitted curves for the variation of electrical failure strain with overlap length (*l*) when the overlap spacing (*w*=3mm) and angle (*θ* = 0°) are kept constant. The green bar represents the electrical failure strain of the platinum-plated TPU fibre mats without edge-locking liquid metal. **c** Fitted curves for the variation of electrical failure strain with angle (*θ*) when the overlap spacing (*w*=3mm) and overlap length (*l*=10mm) are kept constant. The green bar represents the electrical failure strain of the platinum-plated TPU fibre mats without edge-locking liquid metal.


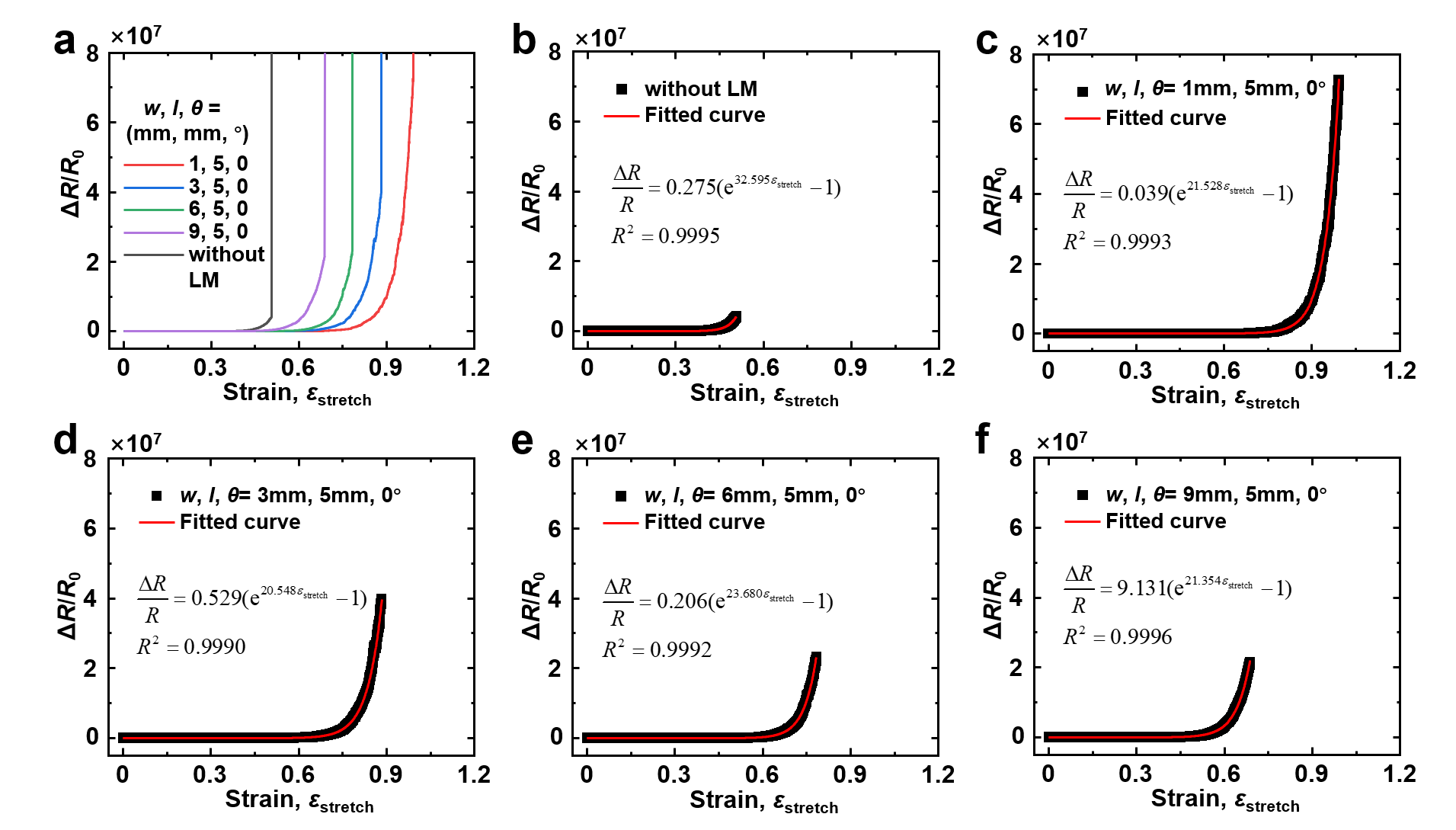


**Supplementary Figure 11. a** Keeping the overlap length (*l*=5mm) and angle (*θ*=0°) constant. Strain sensor response test results obtained by varying the geometry of the patterned liquid metal with overlap spacing (*w*). The black curve is the control without edge-locking liquid metal. **b** Response test results of a platinum-plated TPU fibre mat as a strain sensor without edge-locking liquid metal. The red curves are the fitting results. **c**, **d**, **e** and **f** are the strain sensor response test results obtained when *l*=5mm and *θ*=0° are constant and *w*=1mm, 3mm, 6mm and 9mm respectively. The red curves are the fitting results.


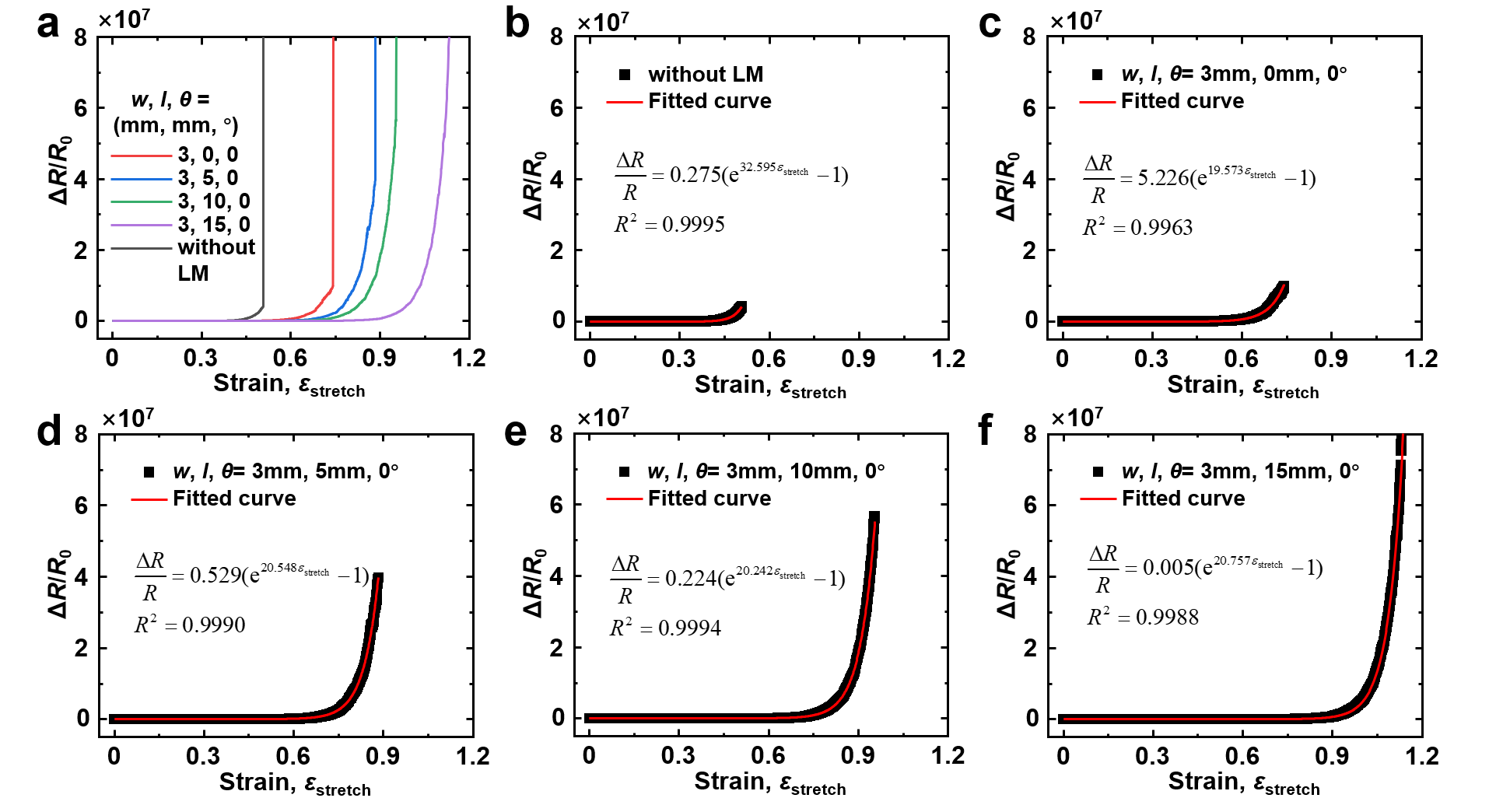


**Supplementary Figure 12. a** Keeping the overlap spacing (*w*=3mm) and angle (*θ*=0°) constant. Strain sensor response test results obtained by varying the geometry of the patterned liquid metal with overlap length (*l*). The black curve is the control without edge-locking liquid metal. **b** Response test results of a platinum-plated TPU fibre mat as a strain sensor without edge-locking liquid metal. The red curves are the fitting results. **c**, **d**, **e** and **f** are the strain sensor response test results obtained when *w*=3mm and *θ*=0° are constant and *l*=0mm, 5mm, 10mm and 15mm respectively. The red curves are the fitting results.


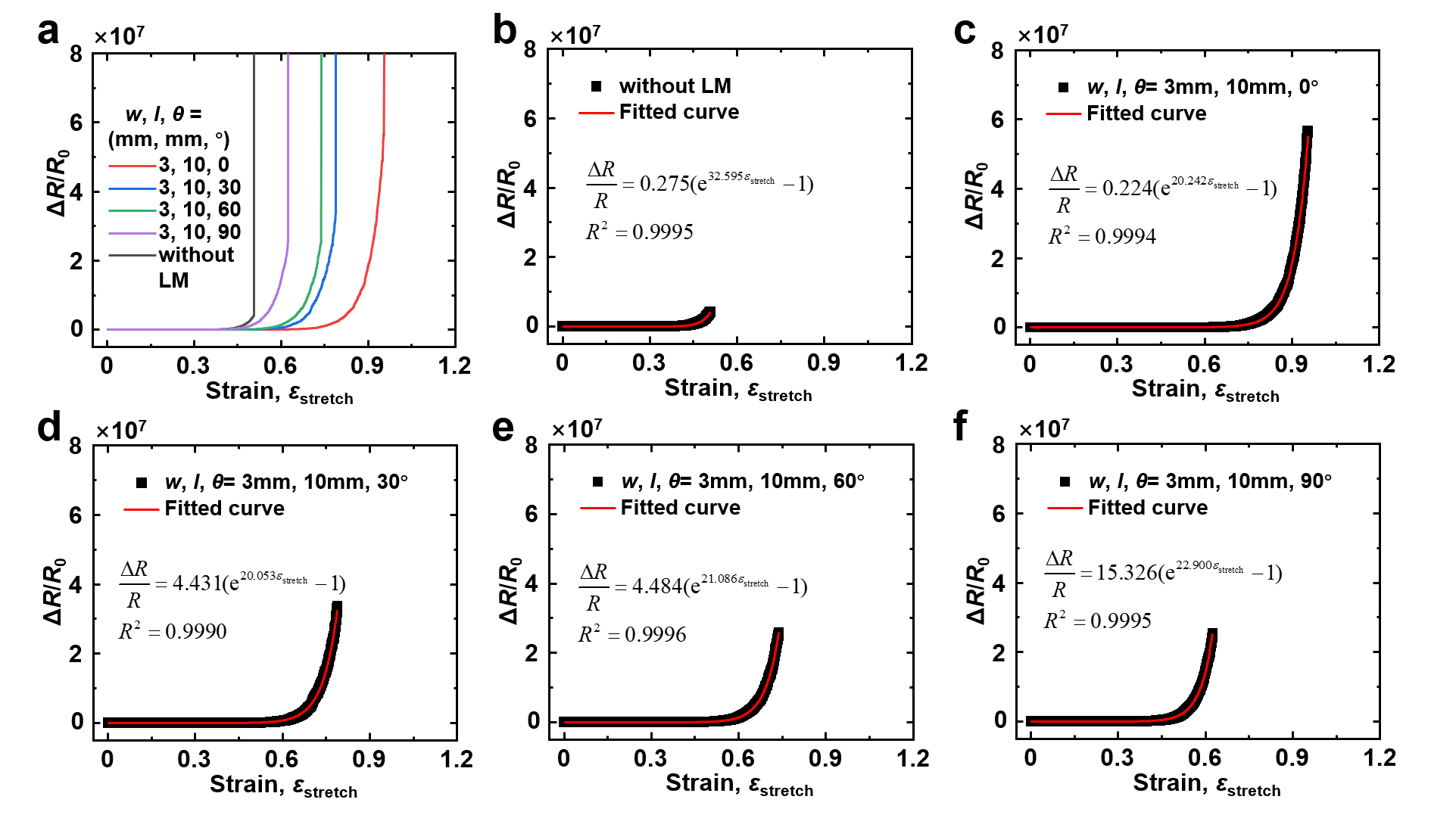


**Supplementary Figure 13. a** Keeping the overlap spacing (*w*=3mm) and overlap length (*l*=10mm) constant. Strain sensor response test results obtained by varying the geometry of the patterned liquid metal with angle (*θ*). The black curve is the control without edge-locking liquid metal. **b** Response test results of a platinum-plated TPU fibre mat as a strain sensor without edge-locking liquid metal. The red curves are the fitting results. **c**, **d**, **e** and **f** are the strain sensor response test results obtained when *w*=3mm and *l*=10mm are constant and *θ*=0°, 30°, 60° and 90° respectively. The red curves are the fitting results.


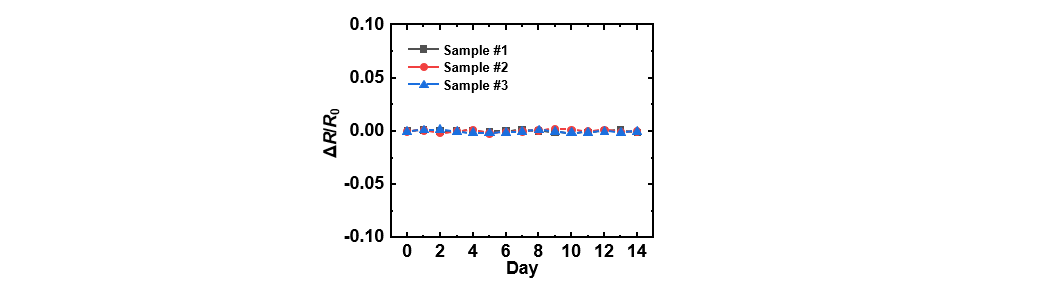


**Supplementary Figure 14.** Resistance change rate of three samples of platinum-plated TPU fibre mats coated with edge-locking liquid metal as strain sensors for 14 consecutive days.


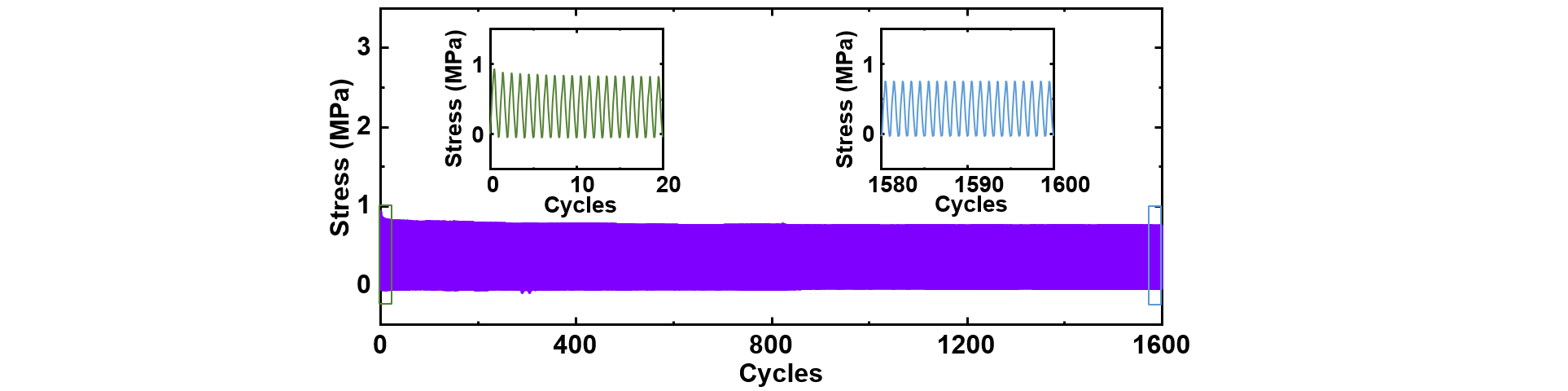


**Supplementary Figure 15.** Cyclic mechanical performance test curves of the sensor.


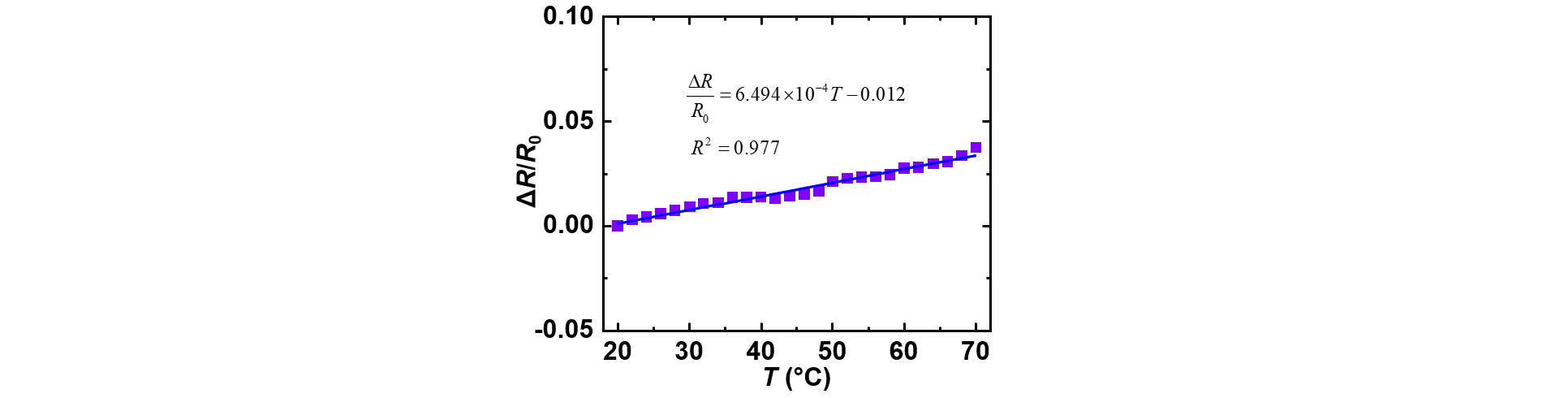


**Supplementary Figure 16.** Temperature response curve of the sensor.


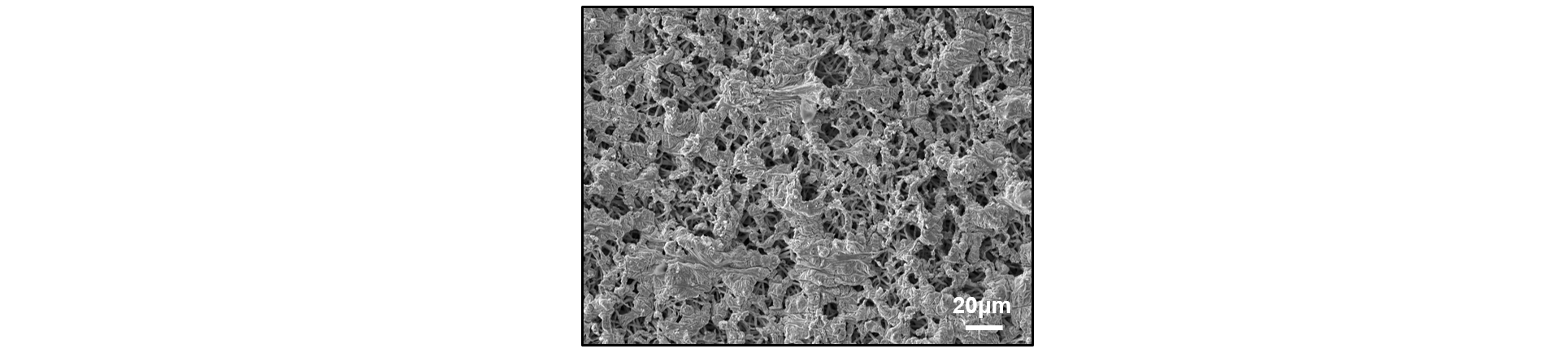


**Supplementary Figure 17.** SEM image of the liquid metal electrode region after 2000 cycles of stretching.


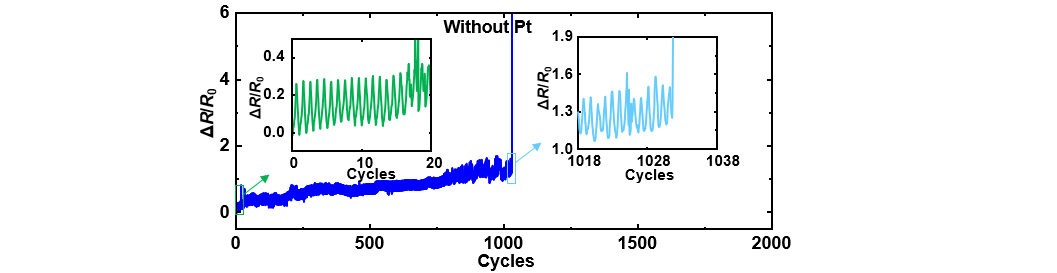


**Supplementary Figure 18.** The long-term electrical response of non-platinum-plated TPU fibre mats coated with liquid metal under 100% cyclic strain at 0.25 Hz for 2000 cycles.


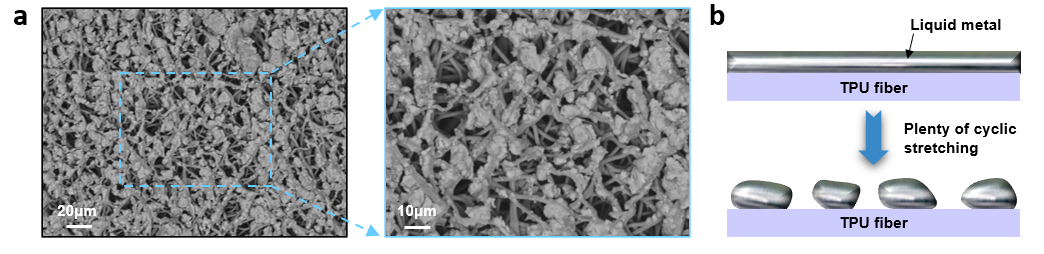


**Supplementary Figure 19. a** SEM image of liquid metal agglomeration leading to electrical failure. **b** Schematic diagram of liquid metal agglomeration after a large number of cycles of stretching following the coating of liquid metal directly on a TPU fibre met.


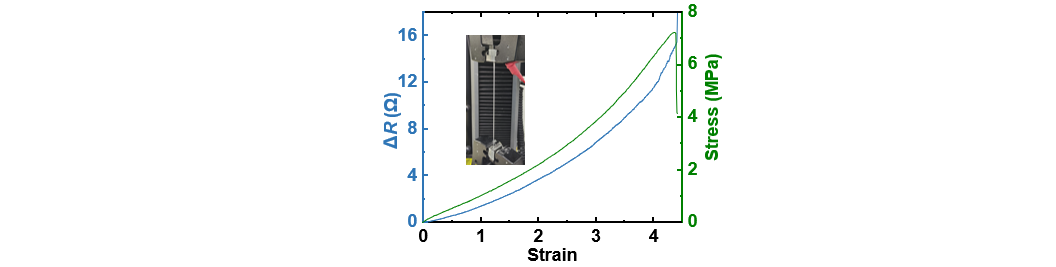


**Supplementary Figure 20.** Variation of resistance (blue curve) and stress (green curve) as a function of strain for platinum-plated TPU fibre mats coated with liquid metal.


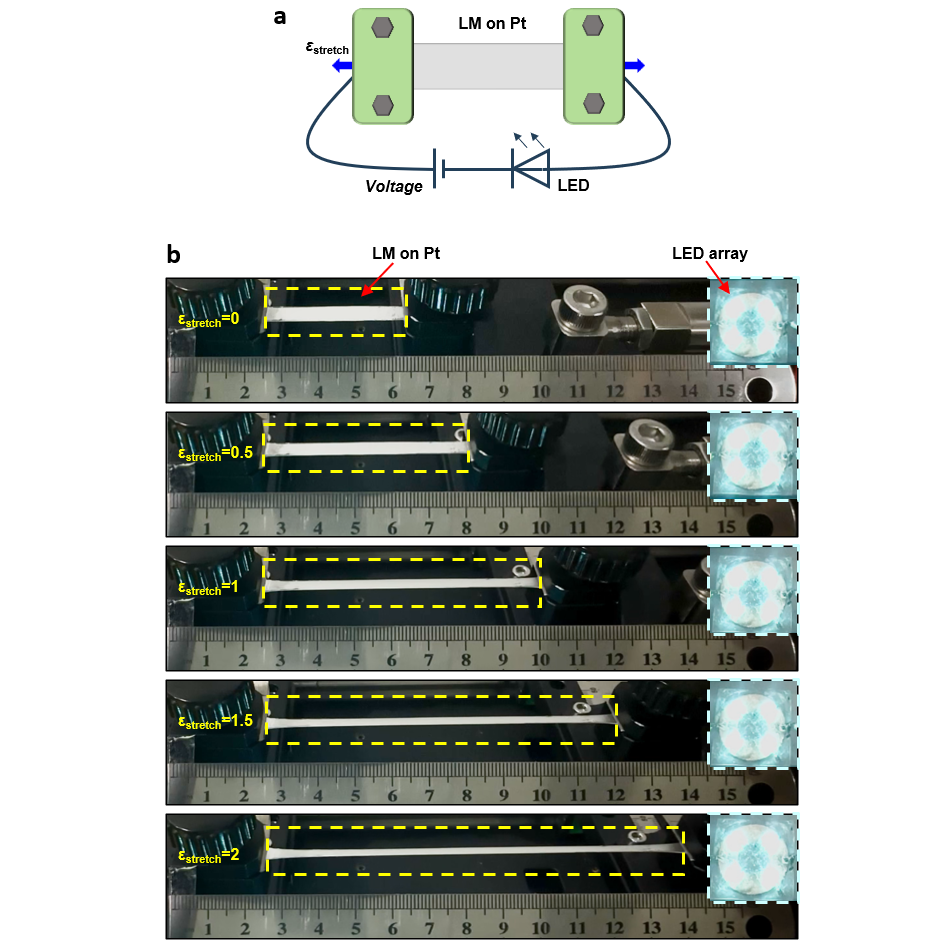


**Supplementary Figure 21. Demonstration of stretchability of platinised TPU fibre mats coated with liquid metal. a** Schematic diagram of a circuit in which a liquid metal-coated platinum-plated TPU fibre felt is used as a piece of wire to connect a light-emitting diode. **b** The diode arrays emit light stably under different strains of the platinum-plated TPU mat coated with liquid metal. From top to bottom, the strains are 0, 0.5,1,1.5,2.


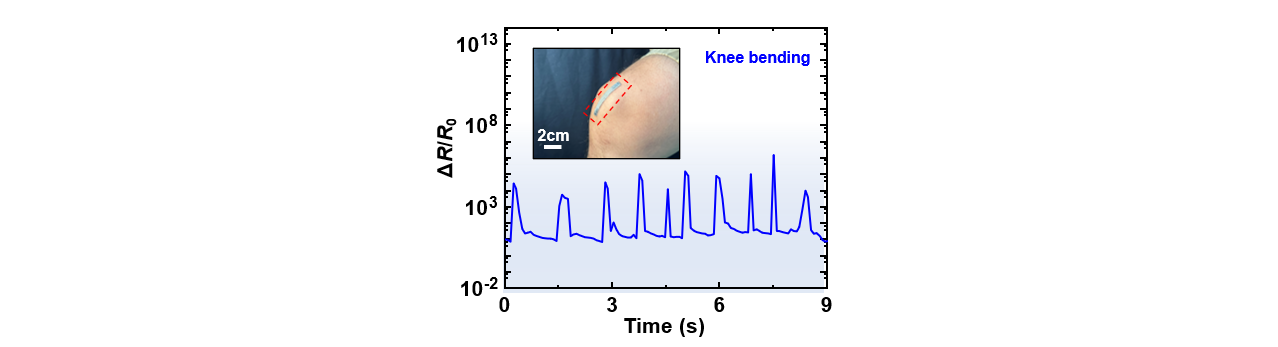


**Supplementary Figure 22.** The resistance change rate of the strain sensor on the knee when doing the step-in-place exercise.

**Supplementary Table 1.** The values of the fitting parameters and the goodness of fit corresponding to the geometrical dimensions of different patterned edge-locking liquid metals.

| ***w*, *l*, *θ***  **(mm, mm, °)=** | ***a*** | ***b*** | ***R*^2^** |
| --- | --- | --- | --- |
| without LM | 0.275 | 32.595 | 0.9995 |
| 3,0,0 | 5.226 | 19.573 | 0.9963 |
| 3,5,0 | 0.539 | 20.548 | 0.9990 |
| 3,10,0 | 0.224 | 20.242 | 0.9994 |
| 3,15,0 | 0.005 | 20.757 | 0.9988 |
| 1,5,0 | 0.039 | 21.528 | 0.9993 |
| 6,5,0 | 0.206 | 23.680 | 0.9992 |
| 9,5,0 | 9.131 | 21.354 | 0.9996 |
| 3,10,30 | 4.431 | 20.053 | 0.9990 |
| 3,10,60 | 4.484 | 21.086 | 0.9996 |
| 3,10,90 | 15.326 | 22.900 | 0.9995 |

**Supplementary Table 2.** Performance statistics of crack or liquid metal based strain sensors in the literature. Green: liquid metal based, orange: crack based, blue: liquid metal and crack based.

| \ | Work range(%) | Gauge Factor | Reference | \ | Work range(%) | Gauge Factor | Reference | \ | Work range(%) | Gauge Factor | Reference | \ | Work range(%) | Gauge Factor | Reference |
| --- | --- | --- | --- | --- | --- | --- | --- | --- | --- | --- | --- | --- | --- | --- | --- |
| 1 | 550 | 0.1 | [1] | 11 | 2 | 2000 | [11] | 22 | 60 | 150000 | [22] | 32 | 82 | 150 | [32] |
| 2 | 450 | 8.333 | [2] | 12 | 1 | 3000 | [12] | 23 | 8 | 10486 | [23] | 33 | 100 | 100 | [33] |
| 3 | 320 | 4.91 | [3] | 13 | 2 | 16000 | [13] | 24 | 20 | 2580 | [24] | 34 | 100 | 7.1 | [34] |
| 4 | 300 | 1.333 | [4] | 14 | 1 | 5000 | [14] | 25 | 0.55 | 2000 | [25] | 35 | 40 | 143 | [35] |
| 5 | 800 | 1.8 | [5] | 15 | 1 | 5000 | [15] | 26 | 20 | 153 | [26] |  | 170 | 30.3 |  |
| 6 | 100 | 1.1877 | [6] | 16 | 83 | 8767.4 | [16] | 27 | 2.5 | 5000 | [27] | 36 | 10 | 1360 | [36] |
| 7 | 400 | 7.16 | [7] | 17 | 130 | 772.6 | [17] | 28 | 100 | 12274 | [28] |  | 79 | 100 |  |
| 8 | 200 | 1 | [8] | 18 | 80 | 2369.1 | [18] | 29 | 500 | 1667 | [29] | 37 | 85 | 2632 | [37] |
| 9 | 105 | 2.2 | [9] | 19 | 200 | 659 | [19] | 30 | 350 | 9.9 | [30] | 38 | 260 | 6.1 | [38] |
| 10 | 500 | 0.5 | [10] | 20 | 110 | 171.06 | [20] | 31 | 6 | 10^3 | [31] | 39 | 25 | 220 | [39] |
|  | 250 | 1.55 |  | 21 | 9.6 | 926 | [21] |  | 7 | 10^6 |  |  | 45 | 90 |  |

[1] Y. Liu, T. Yang, Y. Zhang, G. Qu, S. Wei, Z. Liu, T. Kong, *Advanced Materials* **2019**, 31, 1902783.

[2] J. Wang, S. Yang, P. Ding, X. Cao, Y. Zhang, S. Cao, K. Zhang, S. Kong, Y. Zhou, X. Wang, D. Li, D. Kong, *Acs Applied Materials & Interfaces* **2019**, 11, 18590.

[3] J. Chen, J. Zhang, Z. Luo, J. Zhang, L. Li, Y. Su, X. Gao, Y. Li, W. Tang, C. Cao, Q. Liu, L. Wang, H. Li, *Acs Applied Materials & Interfaces* **2020**, 12, 22200.

[4] D. Kim, Y. Yoon, S. K. Kauh, J. Lee, *Advanced Functional Materials* **2018**, 28, 1800380.

[5] C. A. Silva, J. lv, L. Yin, I. Jeerapan, G. Innocenzi, F. Soto, Y.-G. Ha, J. Wang, *Advanced Functional Materials* **2020**, 30, 2002041.

[6] L.-y. Zhou, J.-z. Fu, Q. Gao, P. Zhao, Y. He, *Advanced Functional Materials* **2020**, 30, 1906683.

[7] H. Wu, H. Qi, X. Wang, Y. Qiu, K. Shi, H. Zhang, Z. Zhang, W. Zhang, Y. Tian, *Journal of Materials Chemistry C* **2022**, 10, 8206.

[8] S. Li, P. Cao, F. Li, W. Asghar, Y. Wu, H. Xiao, Y. Liu, Y. Zhou, H. Yang, Y. Zhang, J. Shang, D. Makarov, R.-W. Li, *Nano Energy* **2022**, 92, 106754.

[9] Y. Wu, Y. Zhou, W. Asghar, Y. Liu, F. Li, D. Sun, C. Hu, Z. Wu, J. Shang, Z. Yu, R.-W. Li, H. Yang, *Advanced Intelligent Systems* **2021**, 3, 2000235.

[10] X. Qi, H. Zhao, L. Wang, F. Sun, X. Ye, X. Zhang, M. Tian, L. Qu, *Chemical Engineering Journal* **2022**, 437, 135382.

[11] D. Kang, P. V. Pikhitsa, Y. W. Choi, C. Lee, S. S. Shin, L. Piao, B. Park, K.-Y. Suh, T.-i. Kim, M. Choi, *Nature* **2014**, 516, 222.

[12] W. S. Lee, D. Kim, B. Park, H. Joh, H. K. Woo, Y.-K. Hong, T.-i. Kim, D.-H. Ha, S. J. Oh, *Advanced Functional Materials* **2019**, 29, 1806714.

[13] B. Park, J. Kim, D. Kang, C. Jeong, K. S. Kim, J. U. Kim, P. J. Yoo, T.-i. Kim, *Advanced Materials* **2016**, 28, 8130.

[14] C. Wang, J. Zhao, C. Ma, J. Sun, L. Tian, X. Li, F. Li, X. Han, C. Liu, C. Shen, L. Dong, J. Yang, C. Pan, *Nano Energy* **2017**, 34, 578.

[15] T. Yang, X. Li, X. Jiang, S. Lin, J. Lao, J. Shi, Z. Zhen, Z. Li, H. Zhu, *Materials Horizons* **2016**, 3, 248.

[16] X. Shi, H. Wang, X. Xie, Q. Xue, J. Zhang, S. Kang, C. Wang, J. Liang, Y. Chen, *Acs Nano* **2019**, 13, 649.

[17] Y. Cai, J. Shen, G. Ge, Y. Zhang, W. Jin, W. Huang, J. Shao, J. Yang, X. Dong, *Acs Nano* **2018**, 12, 56.

[18] M. Chao, Y. Wang, D. Ma, X. Wu, W. Zhang, L. Zhang, P. Wan, *Nano Energy* **2020**, 78, 105187.

[19] J. Lee, S. Shin, S. Lee, J. Song, S. Kang, H. Han, S. Kim, S. Kim, J. Seo, D. Kim, T. Lee, *Acs Nano* **2018**, 12, 4259.

[20] Y. Sun, L. Yang, K. Xia, H. Liu, D. Han, Y. Zhang, J. Zhang, *Advanced Materials* **2018**, 30, 1803189.

[21] Y. Heo, Y. Hwang, H. S. Jung, S.-H. Choa, H. C. Ko, *Small* **2017**, 13, 1700070.

[22] X. Liao, Z. Zhang, Z. Kang, F. Gao, Q. Liao, Y. Zhang, *Materials Horizons* **2017**, 4, 502.

[23] G.-S. Liu, F. Yang, J. Xu, Y. Kong, H. Zheng, L. Chen, Y. Chen, M. X. Wu, B.-R. Yang, Y. Luo, Z. Chen, *Acs Applied Materials & Interfaces* **2020**, 12, 47729.

[24] S. Gong, L. W. Yap, Y. Zhu, B. Zhu, Y. Wang, Y. Ling, Y. Zhao, T. An, Y. Lu, W. Cheng, *Advanced Functional Materials* **2020**, 30, 1910717.

[25] K. K. Kim, I. Ha, M. Kim, J. Choi, P. Won, S. Jo, S. H. Ko, *Nature Communications* **2020**, 11, 2149.

[26] X. Cheng, J. Cai, J. Xu, D. Gong, *Acs Applied Materials & Interfaces* **2022**, 14, 39230.

[27] H. R. Na, H. J. Lee, J. H. Jeon, H.-J. Kim, S.-K. Jerng, S. B. Roy, S.-H. Chun, S. Lee, Y. J. Yun, *Npj Flexible Electronics* **2022**, 6, 2.

[28] K.-H. Kim, S. K. Hong, S.-H. Ha, L. Li, H. W. Lee, J.-M. Kim, *Materials Horizons* **2020**, 7, 2662.

[29] M. Liu, Y. Sheng, C. Huang, Y. Zhou, L. Jiang, M. Tian, S. Chen, S. Jerrams, F. Zhou, J. Yu, *Composites Part A: Applied Science and Manufacturing* **2023**, 164, 107296.

[30] S. Gong, D. T. H. Lai, B. Su, K. J. Si, Z. Ma, L. W. Yap, P. Guo, W. Cheng, *Advanced Electronic Materials* **2015**, 1, 1400063.

[31] X. Li, R. Zhang, W. Yu, K. Wang, J. Wei, D. Wu, A. Cao, Z. Li, Y. Cheng, Q. Zheng, R. S. Ruoff, H. Zhu, *Scientific Reports* **2012**, 2, 870.

[32] Q. Liu, J. Chen, Y. Li, G. Shi, *Acs Nano* **2016**, 10, 7901.

[33] Z. Liu, D. Qi, P. Guo, Y. Liu, B. Zhu, H. Yang, Y. Liu, B. Li, C. Zhang, J. Yu, B. Liedberg, X. Chen, *Advanced Materials* **2015**, 27, 6230.

[34] C. Yan, J. Wang, W. Kang, M. Cui, X. Wang, C. Y. Foo, K. J. Chee, P. S. Lee, *Advanced Materials* **2014**, 26, 2022.

[35] H. Yang, X. Yao, Z. Zheng, L. Gong, L. Yuan, Y. Yuan, Y. Liu, *Composites Science and Technology* **2018**, 167, 371.

[36] S. Gong, L. W. Yap, B. Zhu, Q. Zhai, Y. Liu, Q. Lyu, K. Wang, M. Yang, Y. Ling, D. T. H. Lai, F. Marzbanrad, W. Cheng, *Advanced Materials* **2019**, 31, 1903789.

[37] B. Feng, X. Jiang, G. Zou, W. Wang, T. Sun, H. Yang, G. Zhao, M. Dong, Y. Xiao, H. Zhu, L. Liu, *Advanced Functional Materials* **2021**, 31, 2102359.

[38] G. Li, X. Ma, Z. Xu, Y. Shen, M. Yuan, J. Huang, T. Cole, J. Wei, S. Liu, F. J. I. Han, *Iscience* **2022**, 25, 105495.

[39] L. Mao, T. Pan, J. Guo, Y. Ke, J. Zhu, H. Cheng, Y. Lin, *Nanomaterials* **2022**, 12, 882.
